# CaNRT2.1 Is Required for Nitrate but Not Nitrite Uptake in Chili Pepper Pathogen *Colletotrichum acutatum*

**DOI:** 10.3389/fmicb.2020.613674

**Published:** 2021-01-05

**Authors:** Chia-Chi Kuo, Yung-Chu Lin, Li-Hung Chen, Meng-Yi Lin, Ming-Che Shih, Miin-Huey Lee

**Affiliations:** ^1^Department of Plant Pathology, National Chung Hsing University, Taichung, Taiwan; ^2^Advanced Plant Biotechnology Center, National Chung Hsing University, Taichung, Taiwan; ^3^Agricultural Biotechnology Research Center, Academic Sinica, Taipei, Taiwan

**Keywords:** nitrate transporter, T-DNA insertion, chili pepper, colletotrichum, GPI-anchored, RecQ helicase, pathogenicity

## Abstract

Chili peppers are an important food additive used in spicy cuisines worldwide. However, the yield and quality of chilis are threatened by anthracnose disease caused by *Colletotrichum acutatum.* Despite the impact of *C. acutatum* on chili production, the genes involved in fungal development and pathogenicity in this species have not been well characterized. In this study, through T-DNA insertional mutagenesis, we identified a mutant strain termed B7, which is defective for the growth of *C. acutatum* on a minimal nutrient medium. Our bioinformatics analysis revealed that a large fragment DNA (19.8 kb) is deleted from the B7 genome, thus resulting in the deletion of three genes, including *CaGpiP1* encoding a glycosylphosphatidyl-inisotol (GPI)-anchored protein, *CaNRT2.1* encoding a membrane-bound nitrate/nitrite transporter, and *CaRQH1* encoding a RecQ helicase protein. In addition, T-DNA is inserted upstream of the *CaHP1* gene encoding a hypothetical protein. Functional characterization of *CaGpiP1*, *CaNRT2.1*, and *CaHP1* by targeted gene disruption and bioassays indicated that *CaNRT2.1* is responsible for the growth-defective phenotype of B7. Both B7 and *CaNRT2.1* mutant strains cannot utilize nitrate as nitrogen sources, thus restraining the fungal growth on a minimal nutrient medium. In addition to *CaNRT2.1*, our results showed that CaGpiP1 is a cell wall-associated GPI-anchored protein. However, after investigating the functions of *CaGpiP1* and *CaHP1* in fungal pathogenicity, growth, development and stress tolerance, we were unable to uncover the roles of these two genes in *C. acutatum.* Collectively, in this study, our results identify the growth-defective strain B7 via T-DNA insertion and reveal the critical role of CaNRT2.1 in nitrate transportation for the fungal growth of *C. acutatum*.

## Introduction

Chili peppers, belonging to the genus *Capsicum*, are one of the most important food additives. All growth stages of chili pepper fruits can be affected by anthracnose disease. The typical symptom of this disease on chili pepper fruits is sunken necrotic tissues with concentric rings filled with sporulation structure acervuli. Several *Colletotrichum* species, including *C. gloeosporioides*, *C. capsici*, *C. coccodes*, *C. acutatum*, and *C. boninense*, are reported to cause fruit anthracnose on chili peppers (Than et al., 2008; Tozze et al., 2009). Among them, *C. acutatum* is the dominant pathogen in Taiwan, and infection by this pathogen often results in severe yield losses (Liao et al., 2012a). This pathogen can infect the fruit of chili peppers, bell peppers, and tomatoes, as well as mango leaves (Liao et al., 2012a). Especially, this pathogen forms an especially highly branched infection structure (HBPS) in the cuticle layer of chili pepper fruits during the infection (Liao et al., 2012b). Despite the impact of *C. acutatum* on various hosts, the factors involved in its pathogenicity, HBPS formation and nutrient uptake await to be illustrated.

The establishment of DNA insertional mutagenesis libraries is a valuable way to identify new functional genes. In addition, whole-genome sequencing is fast and affordable now, making the forward genetic approach more reliable to detect the affected genes in mutants. The most widely used technique to generate a random-mutagenesis library in fungi is restriction enzyme-mediated integration (REMI) and *Agrobacterium tumefaciens*-mediated transformation (ATMT) (Meyer et al., 2003; Kuspa, 2006). To perform REMI, a plasmid DNA combined with a specific restriction enzyme are used in protoplast-PEG mediated transformation; therefore, the transformation efficiency is susceptible to the yield and quality of protoplasts (Auyong et al., 2012.). In contrast, ATMT can use various fungal materials for transformation, such as conidia, hyphae, fruiting bodies, and protoplasts (Michielse et al., 2005; Li et al., 2017). In addition, a high percentage of single insertion events has often been observed in the transformants generated by ATMT (Michielse et al., 2005; Kuck and Hoff, 2010), although the chance of multiple insertions was reported too (Lee and Bostock, 2006).

The ATMT system has been established in *Colletotrichum* species such as *C. gloeosporioides, C. lagenarium, C. trifolii, and C. acutatum* (de Groot et al., 1998; Tsuji et al., 2003; Takahara et al., 2004; Talhinhas et al., 2008). Several pathogenicity genes have been successfully identified in *Colletotrichum* species by screening random insertional libraries generated by ATMT. *PacC* encoding a pH-responsive transcription factor was identified in an ATMT mutant of *C. acutatum*, which is a lemon leaf anthracnose pathogen. For instance, *PacC* regulates multiple physiological and developmental processes, thus influencing the pathogenesis of *C. acutatum* (You et al., 2007). MEL1 is a melanin-deficiency gene identified in ATMT mutant T734 of *C. higginsianum*. T734 showed abnormal colonies, albino appressoria, and less virulence on *Arabidopsis* (Liu et al., 2013).

Nitrogen is an essential element of fungus. A fungal cell uptakes organic or inorganic nitrogen-containing compounds and incorporates them into nitrogenous cellular components. For inorganic nitrogen, fungi preferentially use nitrate and ammonium. The mechanisms of the uptake and assimilation of these two types of inorganic nitrogen are not clear in phytopathogenic fungi but have been intensively studied in several model fungi and symbiont fungi. For example, ammonium uptake occurs via a permease in *Penicillium chrysogenum*, *Aspergillus nidulans* (Hackette et al., 1970), and arbuscular mycorrhiza (Calabrese et al., 2016; Ellerbeck et al., 2013), while nitrate uptake occurs by nitrate transporters in *A. nidulans* (Davis and Wong, 2010). *A. nidulans* carries two high-affinity nitrate transporter genes, nrtA and nrtB, for nitrate uptake. If the fungus loses either of these genes, its ability of nitrate uptake is not affected. However, if the fungus loses both genes, it cannot grow on nitrate medium (Unkles et al., 2001). After nitrate is transferred into the cell, it is converted into nitrite and then ammonium by nitrate reductase and nitrite reductase, respectively (Daniel-Vedele et al., 1998).

Although *C. acutatum* is very destructive to many important economic crops, the genes involved in growth and pathogenicity of this fungus remain largely unclear. In this study, we built an *Agrobacterium* T-DNA insertion library and screened for growth defect mutants. Among 198 screened mutants, we identified transformant B7 that lost the ability to grow on Czapek’s medium. B7 contains a T-DNA insertion that causes a 19.8 kb DNA fragment deletion in the genome. Our bioinformatic analysis identified three proteins encoded by this deleted region, including a GPI-anchored protein (CaGpiP1), a membrane-bound nitrate/nitrite transporter (CaNRT2.1) and a RecQ helicase protein (CaRQH1). Additionally, a hypothetical protein (CaHP1) was found in the flanking sequence of T-DNA insertion. To further investigate which gene is response for the growth defect of B7, we generated the gene-disrupted mutants of each gene and examined their functions of fungal growth, nutrient uptake, stress tolerance, and virulence. In conclusion, our results showed that *CaNRT2.1* is an essential gene for nitrate transportation in *C. acutatum* Coll-153 and is responsible for the growth defect of *C. acutatum* B7 on Czapek’s medium.

## Materials and Methods

### Fungal Strains and Plant Materials

*C. acutatum* strain Coll-153 was originally isolated from infected chili pepper fruit in the field (Liao et al., 2012a). All fungal transformants used in this study were generated from Coll-153. Fungal strains were routinely cultured on potato dextrose agar (PDA; Difco) for growth determination and culture preservation. Mycelial plugs of 14-day cultures were preserved at 4°C for short-term storage. For long-term storage, surface mycelia and spores were removed from 14-day cultures and placed in 20% glycerol and stored at −80°C. Seeds of chili pepper cv. “GroupZest” were purchased from a seed company (KNOWN-YOU SEED Co., Ltd.) and grown in a greenhouse. The mature green fruits were collected approximately 30 days after flowering for pathogenicity assays. Green bell peppers and tomato fruits purchased from local supermarkets were used for pathogenicity assays, as well.

### Binary Vector Constructions

All primers and constructed vectors used in this study are listed in Supplementary Tables S1, S2, respectively. For *Agrobacterium*-mediated T-DNA random insertion, the vector p1300-CT74 was constructed. Binary vector pCAMBIA 1300 was used as the backbone vector. Plasmid CT-74 (Lorang et al., 2001) carrying *hptII* and GFP cassettes was digested with *Xho*I and then ligated to *Xho*I/*Sal*I-digested pCAMBIA1300 to generate p1300-CT74. The T-DNA of p1300-CT74 containing the complete plasmid pCT74 would be useful for T-DNA insertion site identification by plasmid rescue if needed.

To disrupt the fungal genes efficiently, split markers combined with DNA homologous recombination via T-DNA transformation was used. Binary vectors p1300-5′-Hyg and p1300-3′-Hyg carrying 5′ and 3′ of *hptII* cassette of pCT74, respectively, were constructed and used to clone the 3′ and 5′ flanking sequences of the target gene. The 5′ and 3′ of the *hptII* cassette of pCT74 were amplified using primer sets pTrpC-*Eco*RI/*Xho*I-5′-Hyg and 3′-Hyg-*Xho*I/*Eco*RI-3′-Hyg, respectively. The PCR products were digested with *Xho*I and *Eco*RI and then ligated to *Xho*I and *Eco*RI-digested pCAMBIA1300.

For CaGPiP1-GFP fusion, the pBHt2 binary vector (Mullins et al., 2001) was used as the backbone vector. A dual-promoter construct from pSilent-Dual 1 (Nguyen et al., 2008) digested with *Sac*I/*Xho*I was cloned into *Sac*I/*Sal*I-digested pBHt2 to generate pBHt2SD, and then one of the dual promoters, the trpC promoter, was removed from pBHt2SD by *Hin*dIII digestion followed by self-ligation, resulting in the pBHt2SD-Pgpd vector. Two plasmids, pPgpdG and pPgpdmidG, were then generated based on pBHt2SD-Pgpd and used to construct different CaGpiP1-GFP fusions. To generate pPgpdG, GFP was amplified from pCT74 with primers GFP_*Xma*I_ApaI_*Xho*I_F2/GFP_nosT_*Hin*dIII_R2 and cloned into pBHt2SD-Pgpd after *Xma*I/*Hin*dIII digestion. To generate pPgpdmidG, GFP amplified with primers GFP_*Bam*HI_*Xho*I_ F/GFP del-taa_*Xma*I_ApaI_R was ligated with pBHt2SD-Pgpd after digestion with *Bam*HI/*Xma*I. Three different constructs of CaGpiP1-GFP fusion were generated using pPgpdG or pPgpdmidG as the cloning vector, including GPI-GFP-I with GFP fused at the 3′ end of *CaGpiP1* to generate the sp-GPI-MP-cs-GFP construct, GPI-GFP-II with GFP inserted between GPI-MP and the cs of CaGpiP1 to generate construct sp-GPI-MP-GFP-cs, and GPI-GFP-III with GFP inserted between the sp and GPI-MP of *CaGpiP1* to generate construct sp-GFP-GPI-MP-cs. Construct GPI-GFP-I was made by cloning *CaGpiP1* ORF amplified using the primers gpi-F-*Spe*I/gpi-del TAA-R-*Xma*I to pPgpdG after digestion with *Spe*I/*Xma*I. Construct GPI-GFP-II was generated by two cloning events—cloning the cs fragment (amplified with primers gpi-CS-F-*Xma*I/Tgpi-R-*Hin*dIII) into the 3′ end of GFP and then cloning the partial *CaGpiP1* ORF amplified with the primer gpi-F-*Spe*I/gpi-delCs-R-*Xho*I in the 5′ end of GFP in pPgpdmidG with digestion by *Xma*I/*Hin*dIII and *Spe*I/*Xho*I, respectively. GPI-GFP-III was constructed by cloning partial *CaGpiP1* ORF amplified with the primers mgpi-F-*Xma*I/Tgpi-R-*Hin*dIII into the 3′ end of GFP in pPgpdmidG with digestion by *Xma*I/*Hin*dIII, and then a synthesized DNA fragment containing Pgpd and sp of CaGpiP1 was cloned into this vector with digestion by *Sac*I/*Xho*I.

For gene complementation, the *npt*II cassette was used as the selection marker, and gene complementation cloning vector pN1300 was generated. In this construction, the *npt*II cassette was obtained from pNC1381Xa (Lee et al., 2010) after digestion with *Eco*RI/*Xho*I and then ligated to *Eco*RI/*Xho*I-digested pCAMBIA1300 to generate pN1300. A complementation fragment containing an approximately 1.5-kb promoter region, ORF and 0.2-kb terminator region was amplified using the primers listed in Supplementary Table S1 and KOD-Neo-Plus polymerase (Toyobo, Japan). The fragment was cloned into pN1300 after digestion with restriction enzymes *Xma*I and *Xba*I.

### *Agrobacterium* T-DNA-Mediated Transformation

*Agrobacterium* T-DNA-mediated transformation was performed as described by Lee and Bostock (Lee and Bostock, 2006) with slight modification. For T-DNA insertional mutant library generation, *A. tumefaciens* EHA105 carrying binary vector p1300-CT74 was cocultured with a spore suspension at 24°C for 2 days. For gene disruption, the spore suspension was mixed with an EHA105 carrying the p1300-5′-Hyg-3′-target and another EHA105 carrying the p1300-3′-Hyg-5′-target, and then the mixture was cocultured at 24°C for 2 days. The cocultured membrane was transferred onto a PDA plate containing 150 μg/ml hygromycin, 50 μg/ml cephalexin and 200 μg/ml cefotaxime (PDA150HCC). For gene complementation, the cocultured membrane was transferred onto PDA containing 400 μg/ml G418 sulfate, 50 μg/ml cephalexin and 200 μg/ml cefotaxime (PDA400GCC). After cultivation for 3–5 days, the membrane was removed, and the putative transformants were transferred onto a PDA150H or PDA350G plate. The putative transformants were purified by single-spore isolation and then PCR assay and/or GFP fluorescence examination for either T-DNA insertion or gene replacement.

### Inverse PCR and Regular PCR

Inverse PCR was performed as described previously (Lee et al., 2010). Briefly, genomic DNA was digested with *Hin*dIII and self-ligated with T4 DNA ligase. The self-ligated DNA was used as the template, and primers HygR2/lac-pro were used to perform inverse PCR. The PCR product was used for the first nested PCR with the primers LB2/M3-reverse, and the resulting PCR product was then used for the second nested PCR using LB3 and T3 as primers. High-fidelity polymerases including KOD-plus (TOYOBO, Japan) and Super-run (Protech, Taiwan) were used in the inverse and nested PCRs. The final PCR product was purified and sequenced.

For regular PCR, including the transformant confirmation and large deletion fragment identification, PCR was performed using the primers listed in Supplementary Table S1. The deletion of a 19.8-kb fragment was identified by a series of PCR treatments. The DNA fragments used for sequencing were amplified using high-fidelity polymerases. A DNA sequence including the deleted fragment and its upstream region was submitted to NCBI (Accession number: MW085781).

### RNA Extraction and Semiquantitative RT-PCR

Mycelia were collected from PDA cultures, and the total RNA was extracted with TRIzol Reagent (life Technologies, United States) (Rio et al., 2010). Complementary DNA was synthesized using oligo d(T) as the primer and MMLV high-performance reverse transcriptase (Epicenter, United States) at 37°C for 1 h. The gene expression level was detected by semiquantitative RT-PCR as described (Lee et al., 2010). The tubulin gene (Accession number: MW073123) was used as the internal control.

### DNA Extraction and Southern Blot Analysis

The fungal strain was transferred from a 5 to 10-day-old PDA culture by using a transfer loop to streak spores and mycelia onto the surface of a new PDA plate topped with a layer of cellophane. After 2 days of incubation, mycelia were collected for DNA extraction using the hexadecyltrimethyl ammonium bromide (CTAB) extraction method (Talbot, 2001; Lee et al., 2010). Genomic DNA (20 μg) was digested with restriction enzymes, electrophoresed in an agarose gel, blotted onto a nylon membrane, and hybridized with a digoxigenin–labeled probe (Yu et al., 2017). The probe was labeled with digoxigenin-11-dUTP during PCR using primers as indicated in Supplementary Table S1. Hybridization and detection using the DIG High Prime DNA Labeling and Detection Starter Kit II (Roche) were conducted according to the instruction manual.

### Microplate Screening

Transformants from the mutant library were cultured on 96-wells plates containing PDA100H and incubated for 4–6 days for sporulation. Sterile water was added into each well (50 μl/well) and mixed well. Twenty microliter of the liquid in each well was then moved to a 96-well plate containing 180 μl sterile water per well to make the inoculum. Five μl of the inoculum was then inoculated into each well of the 96-well plates with PDA, MS (Modified Mathur’s medium: 0.1% yeast extract, 0.1% peptone, 1% sucrose, 0.27% MgSO_4_⋅7H_2_O, 0.27%KH_2_PO_4_ and 1.5% agar) or modified Czapek’s (0.2% NaNO_3_, 0.05% MGSO_4_.7H_2_O, 0.05% KCl, 0.001% FeSO_4_. 7H_2_O, 0.1% K_2_HPO_4_, 10 mM sucrose and 1.5% agar) medium and incubated at 24°C. The Czapek’s medium used in this study contained lower amount of sugar (10 mM sucrose) compared to regular Czapek’s medium (167 mM sucrose).

### Fungal Growth, Sporulation, Spore Attachment, and Germination

Fungal growth was assayed on PDA, PSA (20% potato extract, 2% sucrose and 1.5% agar) and Czapek’s agar media at 24°C. The colony diameter was recorded. Sporulation was measured from PDA cultures 5 days postinoculation under 12 h light/12 h dark at 24°C. Assays of spore germination and attachment were performed using a 96-well plate as described previously (Liao et al., 2012a).

### Cell Wall-Degrading Enzyme Treatment

Spores from each strain were inoculated into a 250-ml flask containing 100-ml PDB (1 × 10^8^ spores/flask). The flasks were incubated at 25°C, 150 rpm for 18 h. Mycelia were collected by filtering through a layer of miracloth and washed twice with distilled water and then once with osmotic buffer (20 mM CaCl_2_, 1.2 M NaCl, and 10 mM sodium phosphate buffer, pH 5.8). One gram of mycelia was treated with lysing enzyme (Sigma L1412) or cellulase (Sigma C1184) at a concentration of 9 mg/ml for 3–4 h. The digestion solution was examined under a fluorescence microscope.

### pH and Nutrient Utilization Assay

To understand the factors in Czapek’s medium that influence the growth of B7, the medium pH, nitrogen source and carbon source were tested for their effects on B7 growth. For the pH assay, Czapek’s medium was adjusted to pH 3.5, 5.0, 7.0, or 9.0 with 0.1 N NaOH or HCl. For nutrient utilization, NaNO_3_ was replaced with KNO_3_, NaNO_2_, KNO_2_, NH_4_Cl (NH_4_)_2_SO_4_, or yeast extract, while sucrose was replaced with glucose in Czapek’s medium. A 5-μl spore suspension was dropped onto the plate center, and the growth was recorded during cultivation.

### Stress Tolerance Assay

To observe whether fungal strains displayed different tolerances to various environmental stresses, fungi were cultured on PDA containing different chemical substances or on PDA under different culture temperatures. For the cell wall integrity test, Congo Red (CR; 100, 200, 300, 400, and 500 ppm), Calcofluor white (CFW; 25, 50, 75, and 100 ppm) and sodium dodecyl sulfate (SDS; 100, 200, and 300 ppm) were used. For the fungicide resistance assay, 200 ppm iprodione, 1 ppm azoxystrobin and 20 ppm tricyclazole were used. For the osmotic stress assay, mannitol, sorbitol and sodium chloride with final concentrations of 0.6 and 1.2 M were tested. A mycelial plug or spore suspension (5 × 10^3^ spores/5 μl) was inoculated onto the centers of different agar media and incubated at 24°C. For the temperature assay, the plates were incubated at 16, 25, or 30°C. The fungal colony diameters were recorded.

### Pathogenicity Assay

The experiments were performed as described previously (Liao et al., 2012a) with slight modifications. Briefly, fruits were drop-inoculated with 5-μl spore suspensions prepared from the wild-type strain and transformants by paired inoculation (Lee et al., 2010). For wounded inoculation, the fruit surface was wounded with a 27-gauge needle to generate a 5 mm-depth wound and then inoculated with a 5-μl spore suspension. Anthracnose lesions were documented at 5–7 days postinoculation (dpi) for pepper fruits (cv. GroupZest) and 5–14 days for tomato fruits, and the lesion sizes were measured with a Spot Image System (Lee et al., 2010).

### Bioinformatics

DNA sequences and their deduced amino acid sequences were analyzed on the NCBI website^1^ using Blastn or Blastp. Functional domains were analyzed using ScanProsite^2^ or the Conserved Domain Search Service of NCBI. The signal peptide sequence was predicted using SignalIP 4.1^3^. The ω site was determined by Gpi-SOM^4^ and big-PI Predictor^5^ (Mazola et al., 2011). The transmembrane domain was detected with TMHMM Server v. 2.0^6^.

### Experimental Design and Statistical Analysis

Experiments were performed at least twice with at least three replicates of each treatment within each experiment, except as indicated otherwise. The significance of differences was determined by a paired *t*-test in the pathogenicity assays and by a *t*-test or Tukey’s HSD test at *P* < 0.05 in other assays using Statistical Package for the Social Sciences software, version 20 (IBM SPSS software).

## Results

### Generation of T-DNA Insertional Mutants

**FIGURE 1:**
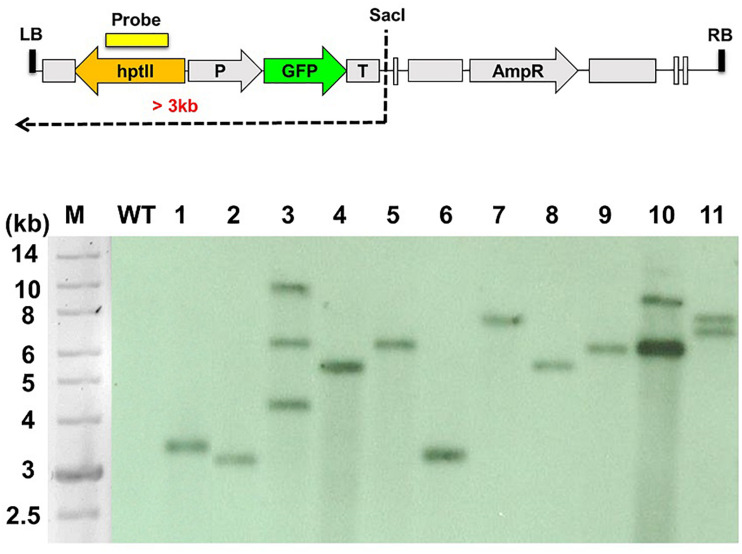
Southern blot analysis of the T-DNA insertion patterns of *Colletotrichum acutatum* Coll-153 transformants. The genetic map of T-DNA used to generate the mutant library is presented in the upper panel. Genomic DNA was digested with *Sac*I and hybridized using *hptII* as the probe. M, DNA molecular marker; WT, wild-type strain; 1-11, randomly selected T-DNA insertion mutants.

*C. acutatum* Coll-153 was transformed with *A. tumefaciens* carrying the binary vector p1300-CT74F. A total of 423 among 490 putative transformants could continually grow on hygromycin amended selection medium. When examined these transformants under a fluorescence microscope, 275 of the 490 transformants expressed intensive green fluorescence. Therefore, the 275 strains were true transformants and were selected for further analysis. The transformation efficiency was 0.0184%, and an average of 5 × 10^4^ spores could yield 9 true transformants. Southern blot analysis of 11 randomly selected T-DNA insertional mutants showed more than 70% of the strains carrying a single T-DNA insertion (Figure 1).

### Identification of the B7 Transformant, a Growth Defect Mutant

**FIGURE 2:**
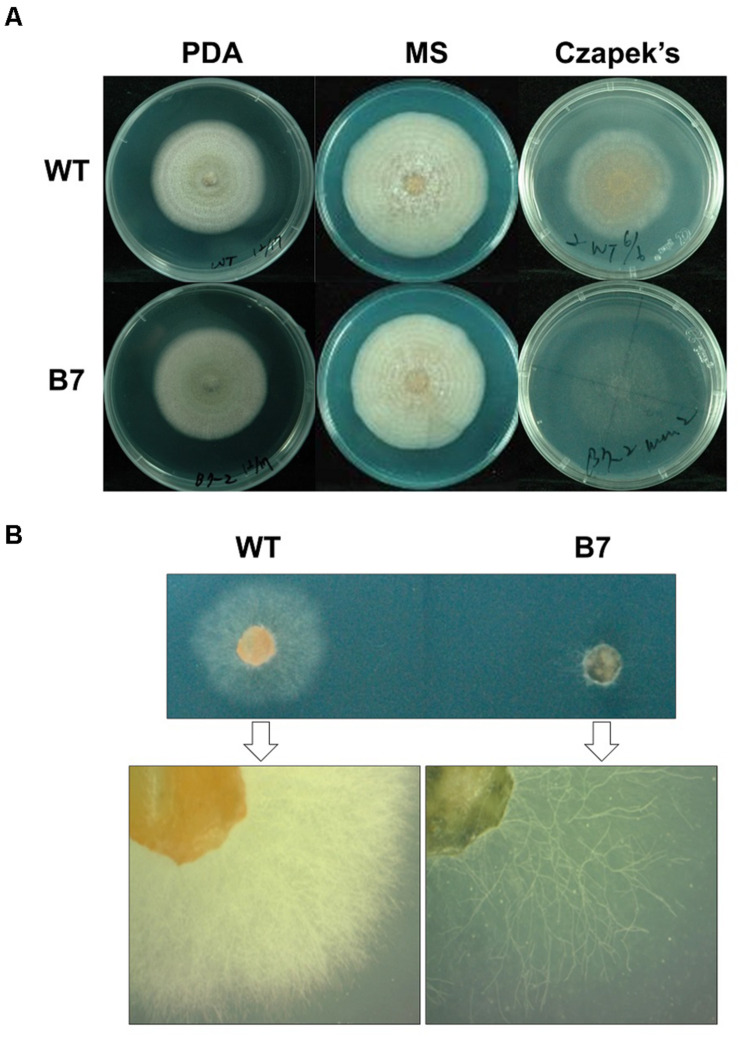
The growth of *Colletotrichum acutatum* Coll-153 strain (wild-type) and transformant B7 on PDA, MS and Czapek’s medium. **(A)** Colony morphology of the wild-type strain (WT) and B7 on PDA (7 days postinoculations), MS agar medium (11 days postinoculations) and Czapek’s agar medium (7 days postinoculation). **(B)** Colonies on Czapek’s agar medium at 3 days postinoculation were examined under a dissecting microscope.

The B7 strain was identified after screening 198 T-DNA insertional mutants for growth defects on a 96-well plate containing Czapek’s medium. The B7 strain hardly grew on Czapek’s medium and showed extremely sparse growth when examined under a dissection microscope. Nevertheless, this growth defect was not observed on the PDA and MS medium (Figure 2). Southern blot analysis indicated that B7 carried a single T-DNA insertion (Supplementary Figure S1). The flanking sequence of the T-DNA left border was cloned to identify the insertion site. Sequence analysis revealed that the T-DNA inserted upstream of the two genes expressed in opposite directions, with one encoding a hypothetic protein (CaHP1) and the other encoding a GPI-anchored protein (CaGpiP1), after blasting against the NCBI database. The expression of the two genes was measured by semiquantitative RT-PCR. The transcript of *CaGpiP1* was not detected in the B7 strain, while *CaHP1* was expressed at a similar level compared to the WT (Supplementary Figure S2).

### The B7 Transformant Lost Its Ability to Use Nitrate as the Sole Nitrogen Source

**FIGURE 3:**
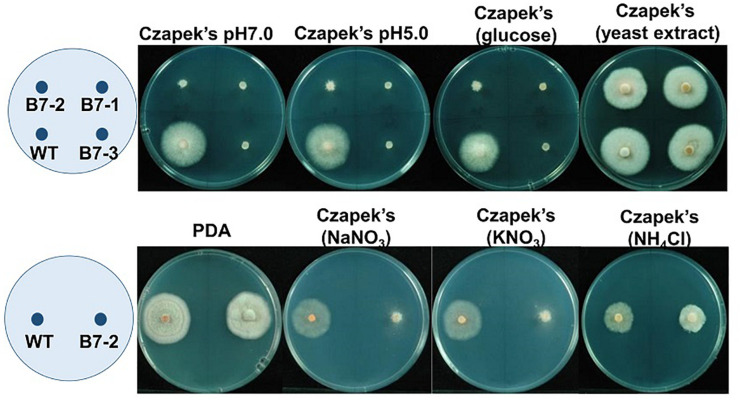
Growth of *Colletotrichum acutatum* wild type (WT) and B7 (B7 single spore isolates B7-1, B7-2 and B7-3) on Czapek’s medium with different pH values (7.0 and 5.0), glucose as the sole carbon source and different sole nitrogen sources (yeast extract, NaNO_3_, KNO_3_ and NH_4_Cl) at 5 days postinoculation. PDA was used as the control treatment.

**FIGURE 4:**
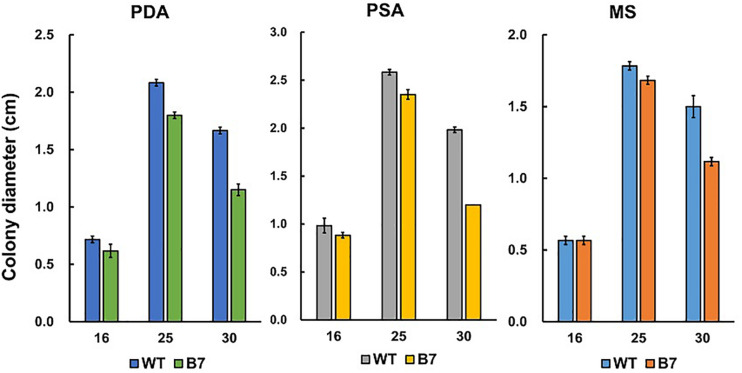
Growth of *Colletotrichum acutatum* wild type (WT) and B7 on PDA, PSA and MS at different temperatures (16, 25, and 30°C).

B7 was found to grow normally on PDA but extreme weakly on Czapek’s medium. Since the pH values of Cazpek’s and PDA media were 7.0 and 5.6, respectively, the pH of Czapek’s medium was adjusted to 5.0 to investigate the effect of the pH on B7 growth. The data indicated that pH was not the factor influencing the growth of B7 on Czapek’s medium (Figure 3). In addition to the pH, nitrogen and carbon sources are two factors that determine fungal growth. Therefore, the effects of the nitrogen and carbon sources on B7 growth were thus investigated. Upon replacing sucrose with D-glucose in Czapek’s medium, no notable difference was observed in B7 growth. However, the growth defect of B7 was recovered when the sodium nitrate in Czapek’s medium was replaced with yeast extract or ammonia chloride but not potassium nitrate, thus indicating that B7 had a defect in the utilization of nitrate (Figure 3). In addition, B7 grew slower compared to the wild type on PDA, PSA and MS media, especially when the culture temperature was raised to 30°C (Figure 4). Further characterization of B7 in terms of pathogenicity and development revealed that B7 displayed similar phenotypes to those of the wild type in virulence, sporulation, and germination (Table 1 and Supplementary Figure S3).

**TABLE 1:** Phenotyping of CaHP1 mutants (Δ*hypo*-4a and Δ*hypo*-3b), CaGpiP1 mutants (Δ*gpi*-11a and Δ*gpi*-B79), CaNRT2.1 mutant (Δnrt-B1), and transformant B7.

**Treatments**		**Strains**					
		**Δ*hypo*-4a**	**Δ*hypo*-3b**	**Δ*gpi*-11a**	**Δ*gpi*-b79**	**Δnrt-B1**	**B7**
Pathogenicity		–^*e*^	–	–	–	–	–
Growth							
	PDA	–	–	–	–	–	Slightly slower growth
	NO_3_^–a^	–	–	–	–	Extremely sparse growth	Extremely sparse growth
	NO_2_^–*a*^	–	–	–	–	–	–
	NH_4_^+*a*^	–	–	–	–	–	–
Spore	Morphology	–	–	–	–	–	–
	Sporulation	–	–	–	–	–	–
	Attachment	–	–	–	–	–	–
	Germination	–	–	–	–	–	–
Stress tolerance							
	Temperature (16 or 32°C)	–	–	–	–	–	Slower growth at 32°C
	Fungicides^*b*^	–	–	–	–	ND	ND^*e*^
	Osmotic^*c*^	–	–	–	–	ND	–
	Cell wall^*d*^	–	–	–	–	ND	ND

*^*a*^Czapek’s medium was used as the basal medium. ^*b*^Fungicides: Iprodione 200 ppm; Azoxystrobin 1 ppm; Tricyclazole 20 ppm; Osmotic stress: 0.6 M and 1.2 M of Mannitol, Sorbitol and NaCl; ^*c*^Cell wall stress: 100–300 ppm SDS, 0–100 ppm Calcofluor white and 0–500 ppm Congo Red. ^*d*^–, no significant difference. ^*e*^ND, not determined.*

### A 19.8-kb Fragment Deletion Occurred in the T-DNA Insertion Region of the B7 Strain

Nitrate utilization genes were suspected to be involved in the growth defect of B7 on Czapek’s medium. Therefore, we searched the nitrate/nitrite transporter gene in the *Colletotrichum* database in NCBI, and we found that a *CaGpiP1* homolog (CH63R_00630) was located in the upstream region of a nitrite transporter gene in *Colletotrichum higginsianum* IMI 349063 when looking at the genomic context. PCR analysis based on the conserved sequences of nitrate/nitrite transporter genes among several *Colletotrichum* species was performed, and sequencing of the PCR product identified the nitrate/nitrite transporter gene (*CaNRT2.1*) in the wild-type strain Coll-153. However, the CaNRT2.1 PCR product could not be amplified from B7. Therefore, a serious of PCR amplifications and sequencings was conducted, and the results showed that a large fragment of DNA had disappeared from the T-DNA insertion site of B7. In total, a 19.8-kb fragment including 3 ORFs (*CaGpiP1*, *CaNRT2.1* and *CaRQH1*) were deleted in B7 (Supplementary Figure S4).

### Bioinformatic Analysis of CaNRT2.1

CaNRT2.1 is a protein with 513 amino acids. Bioinformatic analysis of the functional domains in CaNRT2.1 using the Conserved Domain Search Service of National Center for Biotechnology Information (NCBI) indicated that CaNRT2.1 specifically hit to Major Facilitator Superfamily MFS_1 (pfam07690), and non-specifically hit to the nitrite extrusion protein (accession number TIGR00886), nitrate transmembrane transporter (accession number PLN00028), and MFS_NRT2_like protein (accession number cd17341) that belongs to plant nitrate transporter NRT2 family. Transmembrane domain prediction with the TMHMM Server v. 2.0 discovered 12 transmembrane helices in CaNRT2.1. Analysis by BlastP revealed that the nitrate transporter gene is highly conserved within the *Collectotrichum* genus. The identity of CaNRT2.1 relative to the query proteins was 87–99% with 100% coverage (Supplementary Figure S5). Upon blasting the NCBI protein database with the exclusion of *Colletotrichum* (taxid: 5455), the identity of CaNRT2.1 relative to the query proteins was over 60% with 99% coverage (Supplementary Figure S5), such as 75% to *Verticillium dahliae* and 65% to *Magnaporthe oryzae* 70–15. In addition, it is likely that only one CaNRT2.1 homolog exists within these fungi.

### CaNRT2.1 Restores the Function of Nitrate Utilization in B7

**FIGURE 5:**
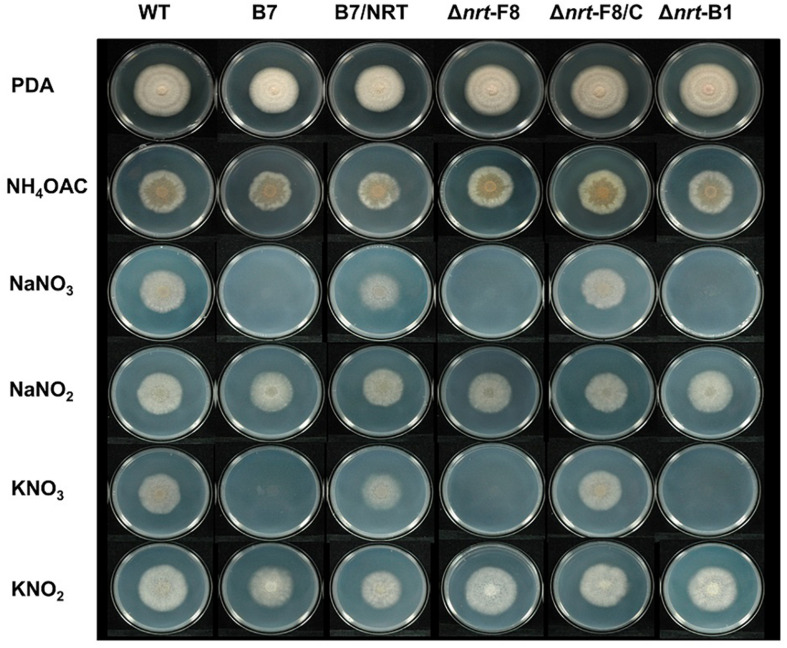
Growth of *Colletotrichum acutatum* wild-type (WT) strain, CaNRT2.1 mutants (Δnrt-F8, Δnrt-B1), CaNRT2.1 complementation strain (Δnrt-F8/C), transformant B7 and B7 complemented with CaNRT2.1 (B7/NRT) on Czapek’s medium containing different nitrogen sources 5 days postinoculation.

**FIGURE 6:**
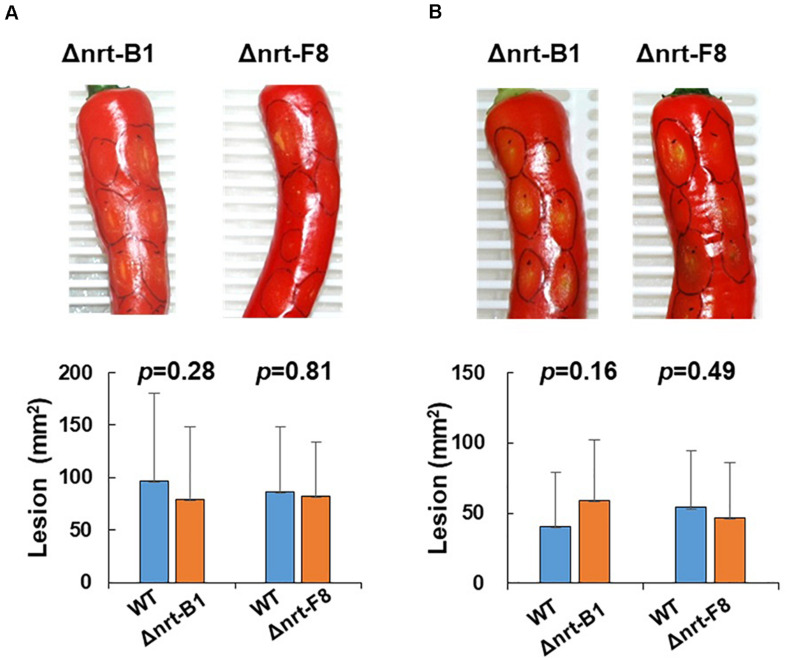
Pathogenicity assay of *Colletotrichum acutatum* Coll-153 wild type (WT) and CaNRT2.1 mutants (Δnrt-B1 and Δnrt-F8) on the fruit of chili pepper cv. GroupZest. All pepper fruits were inoculated with the WT on the left side and a mutant on the right side of a fruit. **(A)** Lesion sizes of fruits 7 days after drop inoculation. **(B)** Lesion sizes of fruits 5 days after wounded inoculation. The means of the lesion sizes are presented in the bottom panels.

To demonstrate that CaNRT2.1 is responsible for nitrate utilization in B7, *CaNRT2.1* was transformed into B7 to generate B7/NRT strains. The transformants were confirmed by PCR assay (Supplementary Figure S6). The growth of the B7/NRT strain was similar to that of the wild-type strain on various nitrogen-containing media (Figure 5). The ability to grow on nitrate media (NaNO_3_ and KNO_3_) was restored in B7 upon gaining the *CaNRT2.1* gene. To further confirm the function of CaNRT2.1 in nitrate utilization, CaNRT2.1-disrupted mutants were generated. Gene replacement of *CaNRT2.1* with the hygromycin resistant gene (*hptII*) was performed with Agrobacterium-mediated T-DNA transformation combined with the split marker strategy. PCR screening for three homologous recombination events occurring within the *hptII* and 5′-flanking region and 3′-flanking region of *CaNRT2.1* identified *CaNRT2.1* gene disruption mutants (strains F8, A1, B1, and A6 in Supplementary Figure S6). The CaNRT2.1-disrupted mutants (Δnrt-F8 and Δnrt-B1) displayed the same growth patterns as those of the B7 strain on various nitrogen media (Figure 5). In addition, the CaNRT2.1-disrupted mutants could use nitrite as the sole nitrogen source, thus indicating that CaNRT2.1 was responsive for nitrate uptake but not for nitrite uptake. Gene complementation strains were generated, and the complementation strain Δnrt-F8/C restored the ability to use nitrate as a nitrogen source (Figure 5 and Supplemental Figure S6). Pathogenicity assays demonstrated no significant difference in lesion sizes caused by the wild-type strain and CaNRT2.1 mutants on chili pepper fruit in both unwounded and wounded treatments (Figure 6). However, the significantly reduced growth of the B7 strain at 30°C was not restored by the complementation of CaNRT2.1 (data not shown).

### Bioinformatics Analysis of *CaHP1*, *CaGPiP1*, and *CaRQH1*

Complementary DNA cloning and sequencing of *CaHP1* and *CaGpiP1* indicated that *CaHP1* encodes a protein with 227 amino acids, and *CaGpiP1* encodes a protein containing 248 amino acids (Supplementary Figures S7, S8). There was no known motif or domain predicted in CaHP1. SignalP analysis revealed a 19-amino acid signal peptide located at the N-terminus of CaGpiP1 (indicated with an underline in Supplementary Figure S8). In addition, the C-terminus of CaGpiP1 was predicted to have a glycosylphosphatidylinositol (GPI) attachment site (ω site) by Gpi-SOM and big-PI Predictor. This indicated that the 23-aa of the C-terminus is a cleavage sequence (CS) that could be removed and replaced with GPI. No additional functional domains were predicted within CaGpiP1. Blasting with the amino acid sequence of CaHP1 and CaGpiP1 against the NCBI database using BlastP indicated that the two proteins were highly conserved in *Collectotrichum* species (Supplementary Figures S7, S8).

Bioinformatics analysis revealed that *CaRQH1* encodes a protein with 1603 amino acids, containing the Superfamilies 1 and 2 helicase ATP-binding type-1 domain (aa723–904), Superfamilies 1 and 2 helicase C-terminal domain (aa914–1078), and HRDC domain (aa1365–1448). Therefore, it was annotated as a RecQ family ATP-dependent DNA helicase (RecQ helicase). Two RecQ helicase genes were found in many *Colletotrichum* species, such as *C. higginsianum* IMI 349063 and *Colletotrichum fioriniae* PJ7, when blasting with the DELTA-BLAST program in BlastP of the NCBI database. This suggests that more than one *CaRQH* gene may exist in the genome of Coll-153.

### Localization of *CaGpiP1* Protein

**FIGURE 7:**
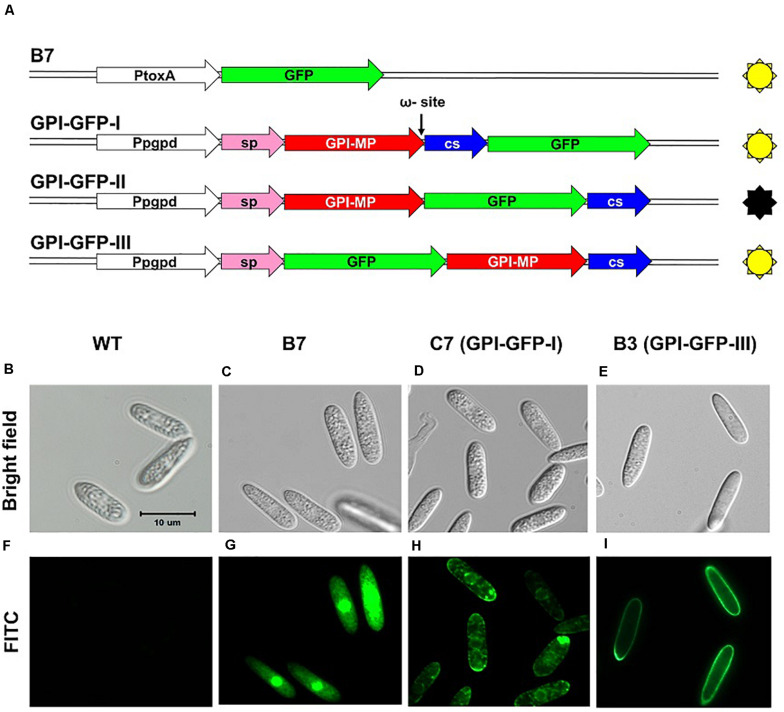
GFP fusion constructs used to examine the localization of CaGpiP1 **(A)** and spore fluorescence images **(B–I)** of transformants generated from different constructs. Conidia from different transformants were examined under a light (bright field, **B–E**) or fluorescence (FITC, **F–I**) microscope. WT, wild-type strain **(B,F)**; B7, transformant B7 **(C,G)**; C7, transformant C7 carrying construct GPI-GFP-I **(D,H)**; B3, transformant B3 with construct GPI-GFP-III **(E,I)**; Pgpd/PtoxA, constitutive promoters; sp, signal peptide; GPI-MP, mature protein of CaGpiP1; CS, cleavage sequence. Bar = 10 μm.

**FIGURE 8:**
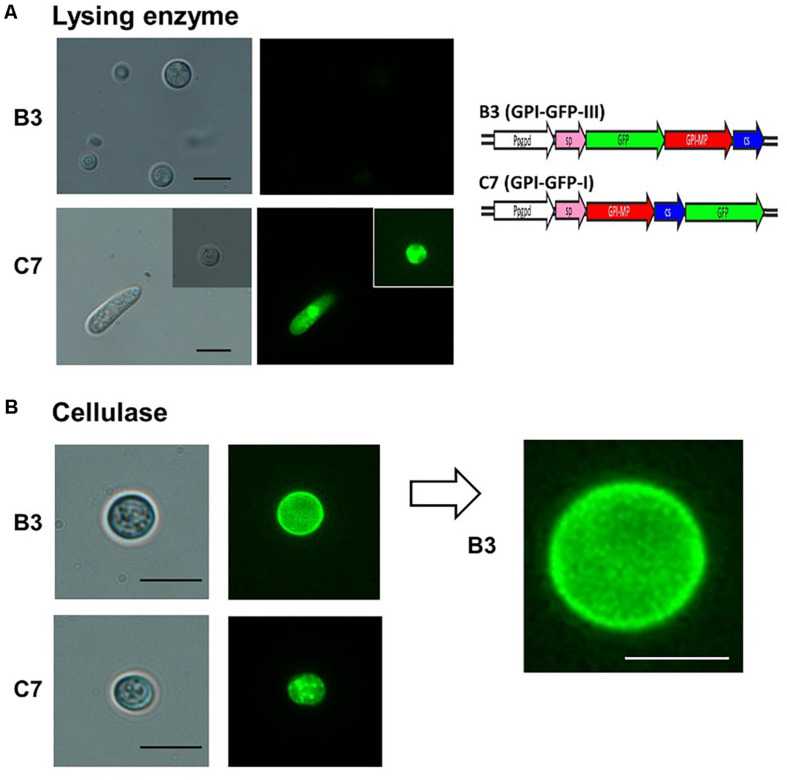
Fluorescence microscopy examination of CaGpiP1 protein localization after digestion with lysing enzyme (Sigma L1412) and cellulose. **(A)** Spores of transformant B3 carrying GPI-GFP-III and transformant C7 carrying GPI-GFP-I were treated with fungal cell wall-degrading enzymes for protoplast formation to examine the localization of CaGpiP1-GFP fusion. Digested cells and undigested cells were examined under a light (left) or fluorescence (right) microscope. **(B)** A cellulase-digested B3 cell was enlarged, and green fluorescent fibers on the cell surface were clearly noticed, which were not seen on the C7 surface. Black bar = 10 μm. White bar = 5 μm.

GPI-anchored proteins were known to be associated with cell membranes or cell walls (Richard and Plaine, 2007); therefore, CaGpiP1 might be involved in fungal development, nutrient uptake and/or stress tolerance. Therefore, we analyzed the localization of CaGpiP1 parallel to the functional analysis of CaGpiP1 by gene disruption. Three binary vectors containing GFP fusions at different positions of CaGpiP1, including GFP fused behind the cleavage site (GPI-GFP-I), in front of the cleavage site (GPI-GFP-II), and behind the signal peptide (GPI-GFP-III), were then constructed and transformed into the wild-type strain of *C. acutatum* via ATMT (Figure 7). Transformants generated from each GFP fusion construct were selected for further experiments. Green fluorescence appeared associated with organelle membranes within the transformant C7 spores carrying GPI-GFP-I. Green fluorescence significantly appeared on the surface of the spores and hyphae in the transformant B3 containing GPI-GFP-III. However, green fluorescence was not observed in the transformants carrying GPI-GFP-II (data not shown). CaGpiP1 and the three GFP fusion proteins (GPI-GFP-I, -II, and -III) were predicted for their cellular localization using PSORT II. The results showed that the GPI-GFP-I fusion protein had equal possibilities of locating at the Golgi body, ER and plasma membrane, while the other two fusion proteins (GPI-GFP-II and -III) and CaGpiP1 had higher possibilities of being secreted or located at the cell wall or cell membrane (Figure 7). To verify the localization of CaGpiP1 in *C. acutatum*, cell-wall degrading enzymes were used to digest the transformants B3 and C7. When B3 and C7 were digested with lysing enzyme, which was a mixture of cellulase, chitinase and protease, green fluorescence was only detected in the protoplasts of C7 but not in those of B3. However, green fluorescence remained on the surface of B3 and within the cell of C7 when the transformants were digested with cellulase. In addition, a fiber-like network with green fluorescence was observed on the cell surface of cellulase-digested B3 but not C7. Collectively, our results indicated that CaGpiP1 was located on the fungal cell wall (Figure 8).

### Functional Analysis of CaHP1 and CaGpiP1

**FIGURE 9:**
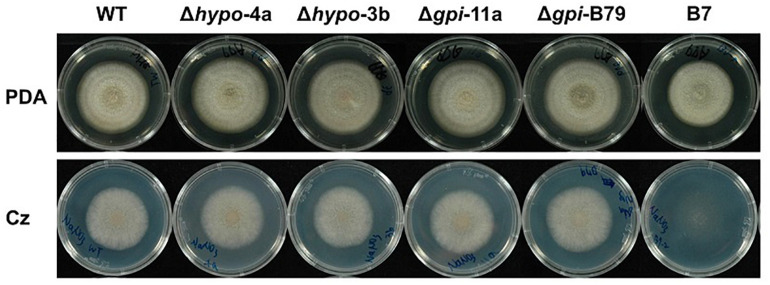
Colony morphology of *Colletotrichum acutatum* wild-type (WT) strain, CaHP1 mutants (Δhypo-4a, Δhypo-3b), CaGpiP1 mutants (Δgpi-11a, Δgpi-B79), and transformant B7 on PDA and Czapek’s medium 6 days postinoculation.

To further investigate the functions of these two genes, gene replacements of *CaHP1* and *CaGpiP1* with hygromycin resistant gene (*hptII*) were generated via ATMT, respectively. The gene-disrupted mutants were verified by PCR assays for three DNA homologous recombination events (Supplementary Figure S9A). Two *CaHP1* mutants and two *CaGpiP1* mutants were verified by Southern blot assays and thus selected for further functional analysis (Supplementary Figure S9B). To understand whether CaHP1 and CaGpiP1 were involved in the growth defect in B7, the gene knockout mutants were assayed on Czapek’s medium containing different nitrogen sources to evaluate the impact of nitrite on the fungal growth. Gene-disrupted mutants of CaHP1 and CgGpiP displayed a normal phenotype compared with the wild-type strain (Figure 9 and Supplementary Figure S10). Thus, the data indicated that CaHP1 and CgGpiP were not involved in nitrate utilization. To investigate the roles of CaHP1 and CgGpiP in fungal pathogenicity, the gene-disrupted mutants were inoculated onto chili pepper, bell pepper, and tomato fruits. The data showed no significant difference in fungal virulence between the wild-type and *CaHP1* and *CaGpiP1* mutants (Supplementary Table S3). Since *CaGpiP1* was located at the cell wall of *C. acutatum* Coll-153, this protein may be involved in environmental stress resistance, as are other fungal GPI-anchored proteins (Plaine et al., 2008; Rittenour and Harris, 2013). Assays of various stress resistances revealed no notable growth difference between WT and CaGpiP1 mutants, including lower (16°C) and higher (30°C) temperatures, fungicide treatments (azoxystrobin, tricyclazole and iprodione), osmotic stress (mannitol, sorbitol and NaCl), and cell wall strength (SDS, Congo red and CFW). No significant difference was detected between the wild type and *CaHP1* mutants in the above assays (Table 1 and Supplementary Figure S11). Additionally, *CaHP1* and *CaGpiP1* mutants also performed similarly to the wild type in sporulation, spore germination, and attachment onto a plastic surface (Table 1 and data not shown).

## Discussion

Effective ATMT transgenic systems have been successfully established in many plant pathogenic fungi to analyze gene function by insertional mutagenesis (Michielse et al., 2005; Maruthachalam et al., 2008, 2011; Huser et al., 2009). T-DNA single insertion without significant deletion of genomic DNA is an important factor in the use of ATMT. In this study, we investigated genes involved in the growth defect of transformant B7 and thus identified a large DNA fragment deletion at the T-DNA insertion site. By gene disruption and functional analysis, we demonstrated that a nitrate transporter encoded by *CaNRT2.1* is responsible for the growth defect of B7 that was unable to use nitrate.

Large DNA fragment deletion caused by T-DNA insertion has been reported in plants since ATMT has been used in plants for several decades. A large-scale survey of published T-DNA insertion lines of *Arabidopsis* reveals that 10% of the T-DNA insertion lines show DNA deletion at the integration site, 73% of which have deletions smaller than 100 bp. The rest of the DNA deletion lines have deletion sizes ranging from 138 to 25-kb, and most of the sizes are smaller than 2 kb (Wang, 2008). The DNA deletion rate caused by T-DNA insertion can reach 87% in *Arabidopsis* (Forsbach et al., 2003). In another study, a 75.8-kb fragment deletion was reported in a T-DNA insertional mutant of *Arabidopsis*, which resulted in the deletion of 15 genes (Kaya et al., 2000). In fungi, ATMT is frequently used for insertional mutagenesis, as in plants (de Groot et al., 1998; Tsuji et al., 2003; Takahara et al., 2004; Talhinhas et al., 2008; Islam et al., 2012). Several studies have performed large-scale analyses of T-DNA insertion patterns in pathogenic fungi, such as *M. oryzae*, *Fusarium oxysporum* f. sp. *lycopersici*, and *Histoplasma capsulatum* (Choi et al., 2007; Meng et al., 2007; Michielse et al., 2009; Kemski et al., 2012). Small genomic deletions at the integration site are frequently detected, and most deletions were less than 30 bp in *M. oyzae* and *H. capsulatum* (Meng et al., 2007; Kemski et al., 2012). The largest deleted fragments reported in these three fungi are 2.6b, 3.4, and 9.8 kb in *M. oryzae*, *H. capsulatum*, and *F. oxysporum* f. sp. *lycopersici*, respectively (Meng et al., 2007; Michielse et al., 2009; Kemski et al., 2012).

When we identified the T-DNA insertion site of the B7 strain, we first thought that a simple insertion with the deletion of a few bases might occur, as we found previously in *Monilinia fructicola* (Lee and Bostock, 2006). However, our PCR assays and bioinformatics results revealed a 19.8-kb fragment deletion in B7. To the best of our knowledge, this is the first report in fungi that demonstrates a deletion fragment larger than 10 kb. Although the mechanisms of DNA deletion caused by T-DNA integration are not yet clear, some studies in Arabidopsis suggest that most deleted fragments were found to carry functions related to DNA replication, repair and mitosis and membrane-bound transporters (Wang, 2008). In addition, genes with high expression levels are frequently affected by T-DNA insertion. In this study, we identified a 19.8-kb deleted fragment in the B7 transformant, which contains three genes encoding a cell wall-localized GPI-anchored protein (CaGpiP1), a membrane-bound nitrate/nitrite transporter (CaNRT2.1) and a RecQ helicase protein (CaRQH1). Among them, RecQ helicase is in one of the helicase families and have a function in DNA replication, recombination, repair, and transcription (Bernstein et al., 2010). Therefore, the function of CaRQH1 might contribute to the large 19.8-kb fragment deletion in B7, as reported in *Arabidopsis* (Wang, 2008).

To examine the function of CaNRT2.1 in the growth defect of B7, we generated gene-disrupted mutants of this gene and conducted functional assays. Our results showed that CaNRT2.1 is involved in nitrate transportation in *C. acutatum*. Nitrate assimilation pathways have been studied well in *Aspergillus nidulans* (Davis and Wong, 2010; Schinko et al., 2010). Both nitrate and nitrite transportation from outside into the cell of *A. nidulans* is dependent on two transporters, NrtA and NrtB, while nitrite can also be absorbed by the cell via transporter NitA (Wang et al., 2008). In the yeast *Hansenula polymorpha*, Ynt1 appears to be the only high-affinity nitrate uptake system, while nitrite uptake in this yeast could be performed by two different transport systems, Ynt1 and a nitrite-specific transporter(s) (Machin et al., 2004). In *Tuber borchii*, a plant symbiotic ascomycete, the nitrate transporter TbNrt2 is bispecific for nitrate and nitrite uptake (Montanini et al., 2006). In this study, our results showed that CaNRT2.1 gene knockout mutants could grow on a medium containing nitrite but not nitrate, indicating that CaNRT2.1 is required for nitrate transportation. In contrast, nitrite transportation is not dependent on CaNRT2.1 in *C. acutatum* strain Coll-153. NCBI blasting with the sequence of *A. nidulans* NitA (AN8674) against the *Colletotrichum* database by BlastX identified homologs in many *Colletotrichum* species (70–82% coverage, 63–70% identity), such as *C. graminicola* M1.001, *C. gloeosporioides* Cg-14 and *C. higgisannum* IMI 349063. This NitA homolog might be a potential transporter for nitrite in *Colletotrichum* species.

CaNRT2.1 belongs to plant nitrate transporter NRT2 family. Plant nitrate transporters are a large family containing three phylogenetically branches (NRT1/PTR, NRT2, and NRT3) (Willmann et al., 2014). The diversification of NRT2 and the origin of its fungal homolog have been illustrated and the results support that eukaryotic NRT2 was from a single origin, the proteobacterial Nitrate/Nitrite Porter (NNP) transporters (Slot et al., 2007). This study also provides five hypotheses to explain the distribution of NRT2 genes in eukaryotes. In conclusion, plants and fungi could gain their NRT2s independently or via horizontal transfer to each other. Plant NRT2s often require partner proteins to transport nitrate. The two-component high affinity nitrate transport system NAR2/NRT2 has been defined in several plant species, in which NAR2 acts as a nitrate accessory protein (Feng et al., 2011). However, whether fungal NRT2 needs the partner protein to promote nitrate uptake appears unclear. Based on the study of nitrate transporter Ynt1 in *H. polymorpha*, it demonstrated that fungal transporter needs the help of the nitrogen permease reactivator 1 kinase (Npr1) for protein phosphorylation to enhance its accumulation in plasma membrane for correct function (Navarro et al., 2008).

In addition to CaNRT2.1, we analyzed CaGpiP1 by examining gene expression levels, function and localization to clarify whether the two genes play roles in the growth defect of B7. CaGpiP1 encodes a protein carrying a GPI-anchoring domain at the C-terminus and a signal peptide located at the N-terminus, but no additional domains were predicted. Gene functional analysis with two gene knockout mutants showed that CaGpiP1 is not involved in nitrite transportation, fungal growth, fungal development, virulence and different stress tolerances including temperature, osmotic, fungicide and cell wall-perturbing compounds. Through GFP-tagging analysis, our data showed that CaGpiP1 localized in the fungal cell wall. Therefore, CaGpiP1 is a function-unknown GpiP located in the cell wall of *C. acutatum*. Although we were unable to find the function of CaGpiP1, this is likely the first GPI-anchored protein that has been analyzed in *Colletotrichum* species. GPI-anchored proteins (GpiPs) are proteins attached by GPI during posttranslation modification and are commonly found in many eukaryotes, such as fungi, plants and mammals. GpiPs contain two conserved regions—an N-terminal signal peptide and a C-terminal GPI anchoring signal. The N-terminal signal peptide guides the GpiP precursor to the endoplasmic reticulum (ER), and the C-terminal GPI anchoring signal of the GpiP precursor is removed when GPI-transamidase links GPI to the cleavage site (ω-site) of GpiP (Boisramé et al., 2011). After posttranslational modification, GpiPs are usually sent to the cell surface. GpiPs show diverse functions depending on the organism (Saha et al., 2016). In fungi, GpiPs have been shown to play roles in cell adhesion, cell wall structure and biosynthesis, and cytotoxicity, but many GpiPs still have unknown functions (Richard and Plaine, 2007; Samalova et al., 2020). In *Saccharomyces cerevisiae*, GpiPs are mainly associated with the cell wall composition and maintenance, flocculation, enzyme activity, sporulation and mating (Pittet and Conzelmann, 2007). In the human pathogen *Candida albicans*, 115 GpiPs were predicted and analyzed, and some of the functions have been speculated to be associated with cell wall synthesis and modification, cell adhesion and the regulation of cell-related interactions or to have enzymatic activity such as superoxide dismutase, though up to 65% of the GpiPs still have no predictable function (Plaine et al., 2008). GpiPs are also required to transport membrane proteins, such as in tryptophan permease in yeasts (Okamoto et al., 2006) and sodium channels in zebrafish (Nakano et al., 2010).

The *CaRQH1* gene encoding a RecQ DNA helicase homolog was also identified in the deleted fragment in B7. RecQ DNA helicase is a highly conserved protein existing in prokaryotes and eukaryotes (Bernstein et al., 2010). RecQ helicase plays a vital role in chromosome stability in fungi (Kato and Inoue, 2006). *Saccharomyces cerevisiae* only has one RecQ homolog, *Sgs1*, in its genome, and the function of Sgs1 has been characterized before. *Sgs1* mutants show an increase in various types of DNA recombination (Myung et al., 2001; Kato and Inoue, 2006), impaired sporulation (Watt et al., 1995) and premature aging of mother cells (Sinclair et al., 1997). By searching the NCBI genome database, five and four RecQ helicase homologs were found in *C higginsianum* IMI 349063 and *C. graminicola* M1.001, respectively, suggesting that multiple Rec Q helicase homologs may have functional overlap to reduce the chances of DNA rearrangement and secure the stability of the chromosome. In addition to the *CaRQH1* gene, *C. acutatum* might also have other RecQ helicase genes to stabilize its genome. B7 and B7/NRT strains show slightly slower growth on the rich media PDA and MS and significantly slower growth on PDA at 30°C compared to the wild type. Slower growth was not observed for CaHP1, CaGpiP1, and CaNRT2.1 mutants. We could not exclude the possibility that CaRQH1 may influence B7 growth on rich media, especially under heat stress. Therefore, the function of CaRQH1 is desired to be illustrated in the future.

In addition to the genes in the deleted fragment, we also identified *CaHP1* in the flanking sequence of T-DNA insertion. *CaHP1* encodes a 227-amino acid protein without any predicted domain. Unfortunately, we were unable to decipher the function of CaHP1 after phenotyping two CaHP1 gene knockout mutants (Δhypo-4a and Δhypo-3b). They displayed similar growth rates to those of the wild type on complex and minimal medium and under low- and high-temperature conditions. They also showed similar abilities in terms of virulence, sporulation, spore germination and adhesion when compared with the wild-type strain. In addition, the further characterization of tolerance to various stresses, as shown in Table 1, reveals that the role of CaHP1 remains unknown.

## Data Availability Statement

The datasets presented in this study can be found in online repositories. The names of the repository/repositories and accession number(s) can be found in the article/Supplementary Material.

## Author Contributions

M-HL, C-CK, and Y-CL contributed to the design of the experiments. Y-CL established the T-DNA insertion library, while C-CK performed gene functional analyses. M-YL was involved in phenotyping of gene disrupted mutants. M-HL and M-CS supervised the experiments. M-HL, L-HC, C-CK, and Y-CL wrote the manuscript. All authors contributed to the article and approved the submitted version.

## Conflict of Interest

The authors declare that the research was conducted in the absence of any commercial or financial relationships that could be construed as a potential conflict of interest.

## Footnotes

1 http://www.ncbi.nlm.nih.gov/

2 https://prosite.expasy.org/scanprosite/

3 http://www.cbs.dtu.dk/s/SignalP/

4 http://gpi.unibe.ch/

5 http://mendel.imp.ac.at/sat/gpi/gpi_server.html

6 http://www.cbs.dtu.dk/services/TMHMM/

## Supplementary Material

Supplementary Figure 1 Southern blot analysis of T-DNA insertion mutant B7. Genomic DNA was digested with *Eco*RI or *Hin*dIII and hybridized using the hptII gene as the probe. Upper panel, Genetic map of T-DNA. Lower panel, Southern blotting image. M, DNA molecular marker (kb); U, undigested genomic DNA; WT, wild-type strain.

Supplementary Figure 2 The localization of T-DNA insertion in transformant B7 (upper panel) and the expressions of the two T-DNA flanking genes analyzed by semiquantitative RT-PCR (lower panel). WT, wild-type strain; Hypo, hypothetical protein; gpi, gpi-anchored protein; tub1, tubulin gene.

Supplementary Figure 3 Pathogenicity assay of *Colletotrichum acutatum* Coll-153 (wild-type, WT) strain and transformant B7 on chili pepper cv. GroupZest. All pepper fruits were inoculated with the WT on the left side and B7 on the right side of a fruit, and lesion sizes were recorded 7 days after drop inoculation.

Supplementary Figure 4 Schematic illustration of the deletion fragment caused by T-DNA insertion **(A)** and multiple PCR assays to identify the deleted region in B7 **(B)**. Primer sets 1–12 were used in the PCR assays. Primer set Tub was used to amplify the tubulin gene for the PCR control. The M13r primer was located at the right border region of the T-DNA. Primers Ntp-3′ck and RecQ-3′ck were located downstream of CaNRT2.1 and CaRecQ1, respectively.

Supplementary Figure 5 Amino acid sequence alignment of CaNRT2.1 homologs. Cn, *Colletotrichum nymphaeae* SA-01 (KXH38191.1); Ch, *Colletotrichum higginsianum* IMI 349063 (XP_018163968.1); Cgr, *Colletotrichum graminicola* M1.001 (XP_008090406.1); Cgl, *Colletotrichum gloeosporioides* Cg-14 (EQB59180.1); Vd, *Verticillium dahliae* VdLs.17 (XP_009649047.1); Mo, *Magnaporthe oryzae* 70-15 (XP_003710887.1). ID, identity; COV, coverage. ID and COV are displayed in the blast results obtained using BlastP against the NCBI protein database.

Supplementary Figure 6 PCR screening for CaNRT2.1 gene replacement mutants. **(A)** CaNRT2.1 complementation transformants of the B7 strain and ΔCaNRT2.1 strain F8 **(B)**. **(A)** Schematic representation of gene replacement with three homologous recombination events using the split marker strategy (upper panel) and PCR assays to confirm that three crossover events occurred in potential CaNRT2.1 gene replaced mutants (F8, A1, B1, A5, and A6). MK, DNA molecular marker; P, plasmid carrying the *hptII* cassette as a positive control of hptII crossover and a negative control of 5′ and 3′ crossover; WT, wild-type strain; N, negative control; tub1 used as the PCR control. Dashed lines indicate the amplified regions of the three crossover fragments. B, PCR amplification of the nptII gene for CaNRT2.1 complementation transformants in B7 and ΔCaNRT2.1 strain F8.

Supplementary Figure 7 Amino acid sequence alignment of CaHP1 homologs. Cs, *Colletotrichum simmondsii* (KXH49087.1); Cf, *Colletotrichum fioriniae* PJ7 (EXF79771.1); Cn, *Colletotrichum nymphaeae* SA-01 (KXH47772.1); Ch, *Colletotrichum higginsianum* IMI 349063 (XP_018163966.1); Cgr, *Colletotrichum graminicola* M1.001 (XP_008090403.1); Vl, *Verticillium longisporum* (CRK19371.1). ID, identity; COV, coverage. ID and COV are displayed in the blast results obtained using BlastP against the NCBI protein database.

Supplementary Figure 8 Amino acid sequence alignment of CaGpiP1 homologs. Signal peptides and cleavage sequences are indicated with boxes, while the ω site is indicated with an arrow. Ca, CaGpiP1; Cn, *Colletotrichum nymphaeae* SA-01 (KXH47773.1); Cf, *Colletotrichum fioriniae* PJ7 (EXF79770.1); Co, *Colletotrichum orbiculare* MAFF 240422 (TDZ25850.1); Cgl, *Colletotrichum gloeosporioides* Cg-14 (EQB59181.1); Cgr, *Colletotrichum graminicola* M1.001 (XP_008090404.1); Vl, *Verticillium longisporum* (CRK19369.1). ID, identity; COV, coverage. ID and COV are displayed in the blast results obtained using BlastP against the NCBI protein database.

Supplementary Figure 9 Screening of CaHP1 and CaGpiP1 gene knockout mutants by PCR **(A)** and Southern blot **(B)** analysis. **(A)** Selection marker gene (*hpt*II), 5′ and 3′ recombination fragments were amplified with primer set P3 and P4, P1 and P2 primers, and P3 and P5, respectively. The full length (FL) of the gene replaced fragment was amplified by primer set P1 and P5. Tubulin1 gene (*tub1*) was used as the positive control in PCR. **(B)** Southern blot analysis was performed using *CaHP1*, *CaGpiP1*, or *hpt*II as the probe. The expected DNA sizes in the Southern bolting assays for the wild type and mutants hybridized with the three probes are indicated below the blotting.

Supplementary Figure 10 Colony morphology of the *Colletotrichum acutatum* wild-type (WT) strain, CaHP1 mutants (Δhypo-4a, Δhypo-3b), CaGpiP1 mutants (Δgpi-11a, Δgpi-B79), and transformant B7 on PDA and Czapek’s medium containing different nitrogen sources at 5 days postinoculation.

Supplementary Figure 11 Growth of *Colletotrichum acutatum* Coll-153 strain (WT), CaHP1 mutants (Δ*hypo*-4a, Δ*hypo*-3b), and CaGpiP1 mutants (Δ*gpi*-11a, Δ*gpi*-B79) under different stresses. Upper panel, colony diameter of fungal strains on PDA supplemented with azoxystrobin, tricyclazole or iprodione at 7 days postinoculation. Bottom panel, colony diameter of fungal strains on PDA amended with NaCl, mannitol, sorbitol, Congo red, or SDS at 5 days postinoculation.

Supplementary Table 1 Primers used in this study.

Supplementary Table 2 Plasmids used in this study.

Supplementary Table 3 The *p*-values of the statistical analysis of the lesion size caused by gene knockout strains of CaHP1 (Δ*hypo*-4a and Δ*hypo*-3b) and CaGpiP1 (Δ*gpi*-11a and Δ*gpi*-B79) or transformant B7 on the fruits of chili pepper, tomato and bell pepper compared with the wild-type strain.
