# Supplementary material for: CaNRT2.1 Is Required for Nitrate but Not Nitrite Uptake in Chili Pepper Pathogen Colletotrichum acutatum
Source: Front Microbiol. 2021 Jan 5;11:613674. doi: 10.3389/fmicb.2020.613674 (PMC7813687; doi:10.3389/fmicb.2020.613674)
Supplement: Supplementary Figure 1 — Southern blot analysis of T-DNA insertion mutant B7. Genomic DNA was digested with EcoRI or HindIII and hybridized using the hptII gene as the probe. Upper panel, Genetic map of T-DNA. Lower panel, Southern blotting image. M, DNA molecular marker (kb); U, undigested genomic DNA; WT, wild-type strain. [file Data_Sheet_1.pdf]

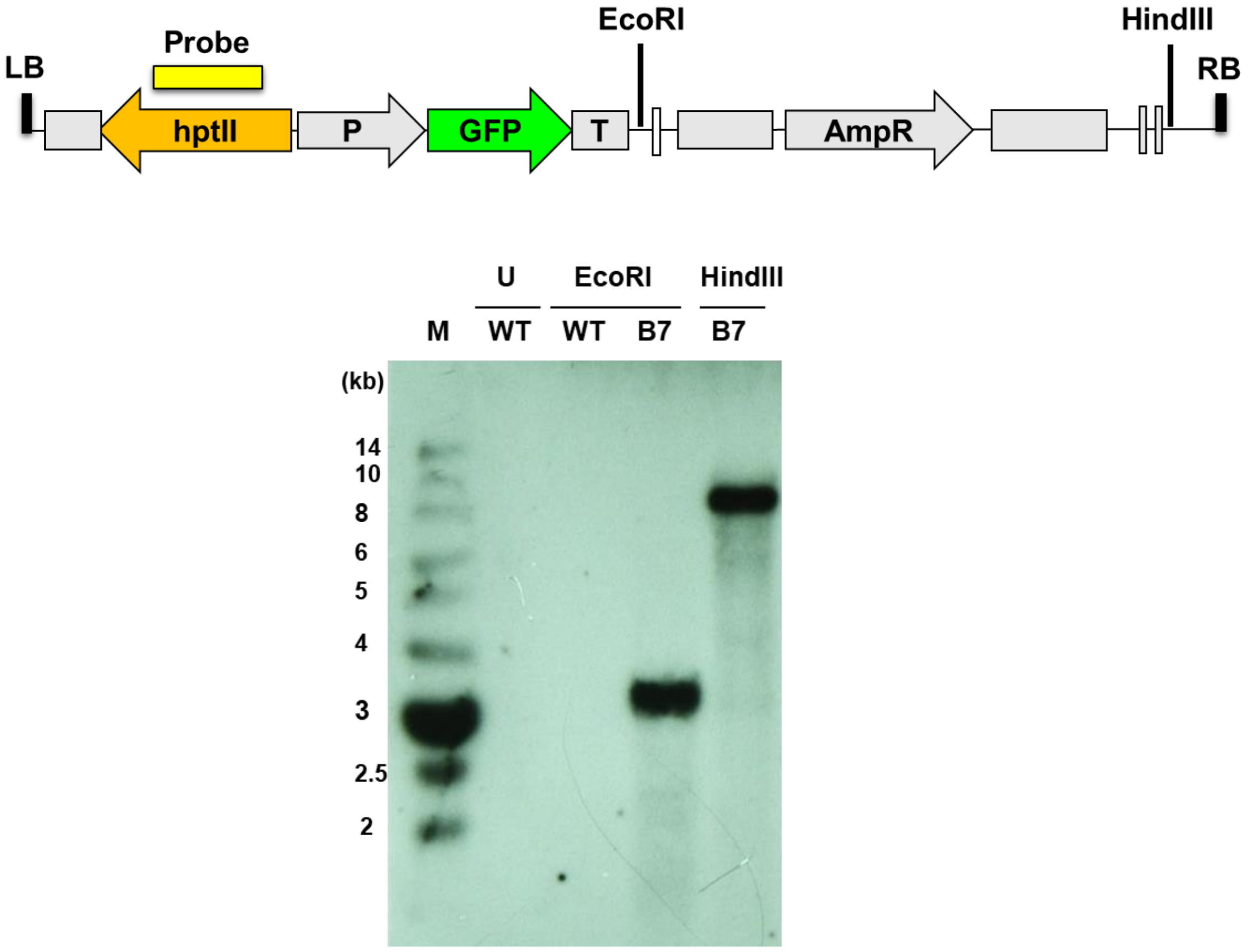

Figure S1. Southern blot analysis of T-DNA insertion mutant B7. Genomic DNA was digested with EcoRI or HindIII and hybridized using the hptII gene as the probe. Upper panel, Genetic map of T-DNA. Lower panel, Southern blotting image. M, DNA molecular marker (kb); U, undigested genomic DNA; WT, wild-type strain.

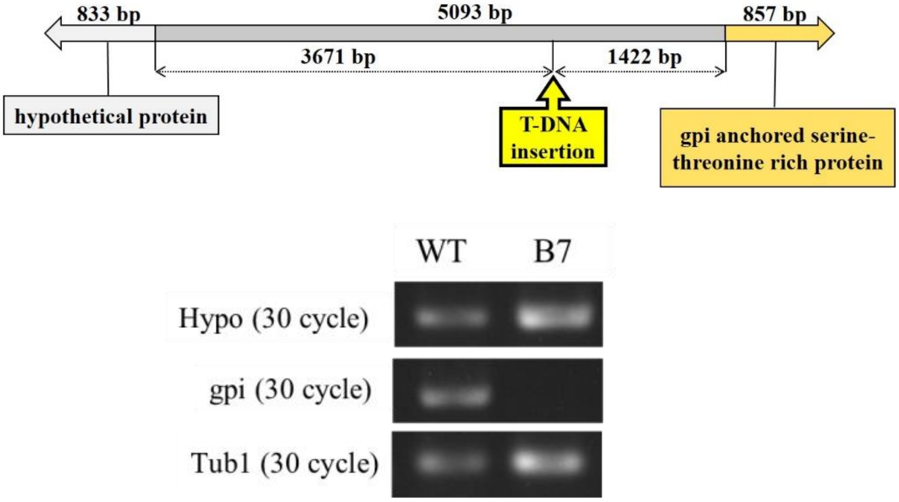

Figure S2. The localization of T-DNA insertion in transformant B7 (upper panel) and the expressions of the two T-DNA flanking genes analyzed by semiquantitative RT-PCR (lower panel). WT, wild-type strain; Hypo, hypothetical protein; gpi, gpi-anchored protein; tub1, tubulin gene.

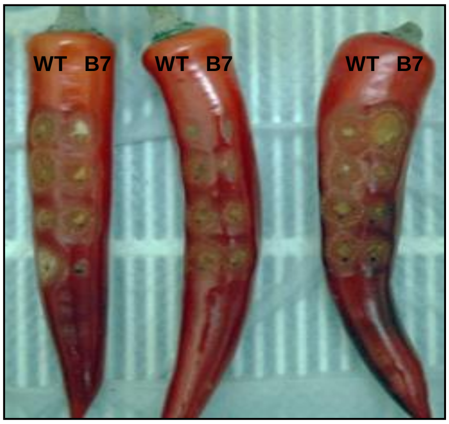

Figure S3. Pathogenicity assay of *Colletotrichum acutatum* Coll-153 (wild-type, WT) strain and transformant B7 on chili pepper cv. GroupZest. All pepper fruits were inoculated with the WT on the left side and B7 on the right side of a fruit, and lesion sizes were recorded 7 days after drop inoculation.

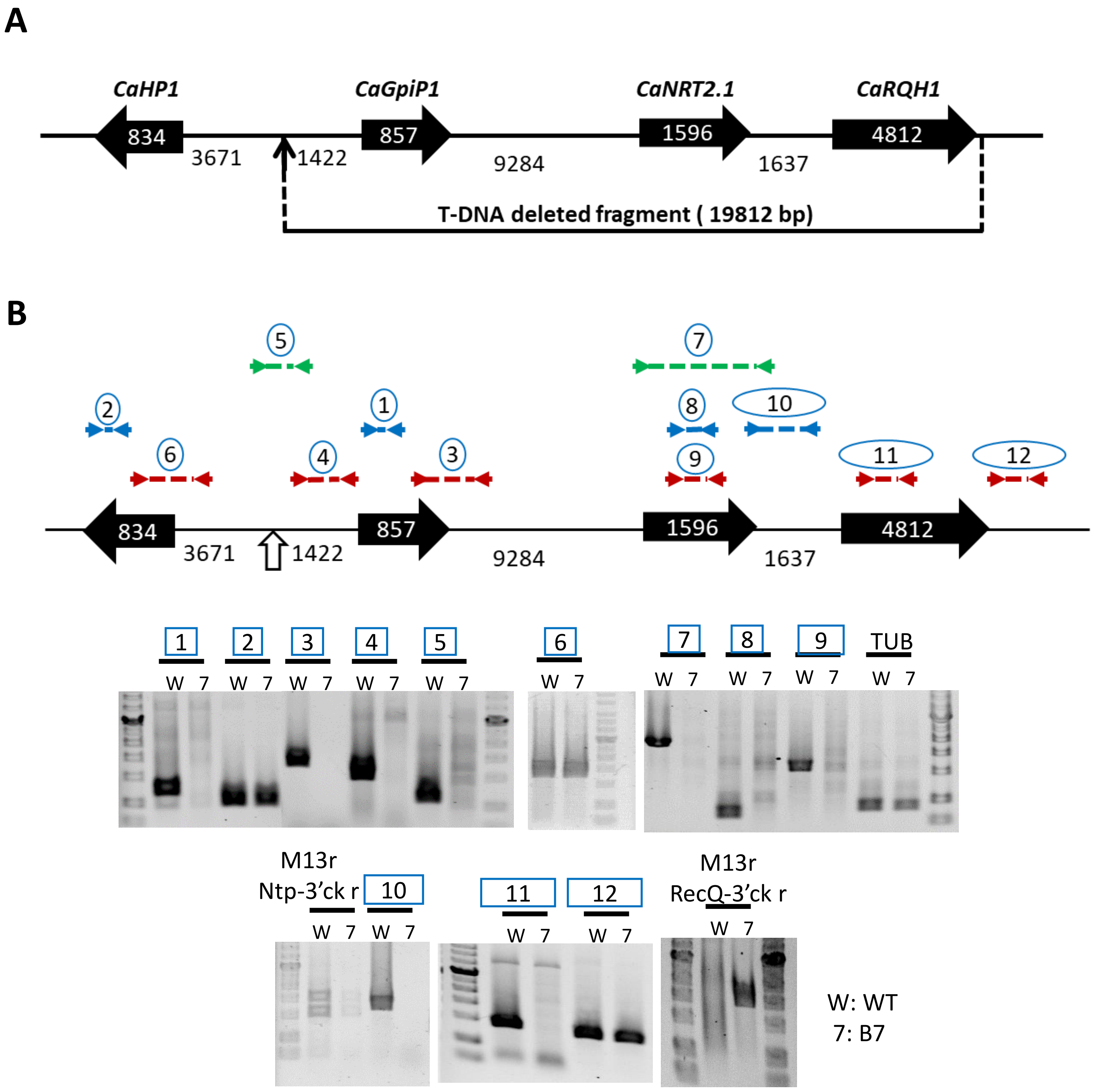

Figure S4. Schematic illustration of the deletion fragment caused by T-DNA insertion (A) and multiple PCR assays to identify the deleted region in B7 (B). Primer sets 1-12 were used in the PCR assays. Primer set Tub was used to amplify the tubulin gene for the PCR control. The M13r primer was located at the right border region of the T-DNA. Primers Ntp-3'ck and RecQ-3'ck were located downstream of CaNRT2.1 and CaRecQ1, respectively.

```
                            10        20        30        40        50        60        70        80
                    ....|....|....|....|....|....|....|....|....|....|....|....|....|....|....|....|
Ca>CaNRT2.1         ----MQLSVLWRAPEVNPINRKARSIPVFNIFNVYARAFHFSWLGFMIAFWAWYTFPPLLTVTIKKDLNLTTAQVANSNI
Cn>KXH38191.1       ----MQLSILWRAPEVNPINRKARSIPVFNIFNVYARAFHFSWLGFMIAFWAWYTFPPLLTVTIKKDLNLTTAQVANSNI
Ch>XP_018163968.1   ----MQLSVLWRAPEVNPVNRKARSIPVLNIFNVYARAFHFSWLGFMIAFWAWYTFPPLLTVTIKKDLKLTNAQVANSNI
Cgr>XP_008090406.1  ----MQLSVLWRAPEVNPINRKARSIPVFNIFNIYARAFHFSWLGFMIAFWAWYTFPPLLTVTIKKDLKLNNAQVANSNI
Cgl>EQB59180.1      ----MELSILWRAPEVNPVNRKARSIPVFNVFNVYARAFHFSWLGFMIAFWAWYTFPPLLTVTIKKDLNLTTAQVANSNI
Vd>XP_009649047.1   ----MKISTLWRAPEVNPITLKARSVPVLNPFNKYGRVFLFSWLGFMIAFWAWYTFPPLLTVTIRKDLALTPQQVANSNI
Mo>XP_003710887.1   MGINVKFSDLYRAPEVNPITRKARSIPALNVINMYGRVFFFSWFGFMIAFWAWYTFPPLLTVTIRKDLNLTAAEVANSNI
Consensus               :::* *:*******:.  ****:*.:* :* *.*.* ***:********************:*** *.  :******

                            90       100       110       120       130       140       150       160
                    ....|....|....|....|....|....|....|....|....|....|....|....|....|....|....|....|
Ca>CaNRT2.1         VSLVATLFMRLIAGPACDRFGSRIVFGSLLLIGTIPIGLAPLVKDATGLYISRFFIGVLGATFVPCQVWCTGFFDKNVVG
Cn>KXH38191.1       VSLVATLFMRLIAGPACDRFGSRIVFGSLLLIGTIPIGLAPLVKDATGLYISRFFIGVLGATFVPCQVWCTGFFDKNVVG
Ch>XP_018163968.1   VSLVATLFMRLIAGPACDRFGSRIVFGSLLLIGTLPIGLAPLVTNATGLYISRFFIGVLGATFVPCQVWCTGFFDKNVVG
Cgr>XP_008090406.1  VSLVATLFMRLIAGPACDRFGSRIVFGSLLLIGTLPVGLAPLVTNVTGLYISRFFIGVLGATFVPCQVWCTGFFDKNVVG
Cgl>EQB59180.1      VSLVATLFMRLIAGPACDRFGSRIVFGSLLLIGSIPIGLAPLVKDATGLYISRFFIGVLGATFVPCQVWCTGFFDKNVVG
Vd>XP_009649047.1   VSLSSTLLMRVIAGPACDKFGSRWVFGGMLLLGSLPIGLAPLVNSANGLYVSRFFIGILGGTFVPCQVWCTGFFDKNIVG
Mo>XP_003710887.1   VSLVATLFVRMVAGPLCDLWGSRVVFGGVLLVGAIPLGLAPLIQNATGLYVSRFFIGILGGAFVPCQVWSTGFFDKNVVG
Consensus           *** :**::*::*** ** :*** ***.:**:*::*:*****: ...***:******:**.:*******.*******:**

                           170       180       190       200       210       220       230       240
                    ....|....|....|....|....|....|....|....|....|....|....|....|....|....|....|....|
Ca>CaNRT2.1         TANALAGGWGNAGGGITYFVMPAVYDSFVHARGYSPGQAWRLTFIVPVICLFCCGMGLLLLCEDTPTGKWADRHLHAQEN
Cn>KXH38191.1       TANALAGGWGNAGGGITYFVMPAVYDSFVHARGYSPGQAWRLTFIVPVICLFCCGMGLLLLCEDTPTGKWADRHLHAQEN
Ch>XP_018163968.1   TANALAGGWGNAGGGITYFVMPAVYDSFVHARGYSPGQAWRLTFIVPVICLFVCGLGLLLLCEDTPTGKWADRHLHAQEN
Cgr>XP_008090406.1  TANALAGGWGNGGGGLSYWVMPAVFDSFVHARGYSPGQAWRMTFIVPVICLLACGLGLLLLCKDTPTGKWADRHLHAQEN
Cgl>EQB59180.1      TANALAGGWGNAGGGITYFVMPAVYDSFVHARGYSPGQAWRLTFIVPVICLLACGLGLLLLCEDTPTGKWADRHLHAQEN
Vd>XP_009649047.1   TANALAGGWGNAGGGVTYFVMPAVFDAFVS-RGYKPGVAWRLTFIVPLICIITCGIALLLLCDDTPTGKWSDRHLHVQEN
Mo>XP_003710887.1   TANALTGGFGNAGGGITYFIMPAVFDSFVHRMGYTPGQAWRLTFVVPLVMIIVTGVSLLLLCPDTPTGKWSERHMHAQQM
Consensus           *****:**:**.***::*::****:*:**    **.** ***:**:**::  ::   *:.***** *******::**:*.*:

                           250       260       270       280       290       300       310       320
                    ....|....|....|....|....|....|....|....|....|....|....|....|....|....|....|....|
Ca>CaNRT2.1         L-HSHGVDAAVRSDVVDVPGGITDRN--NTGSMASD---EEKNSTH-----GKNGVVSDHEAS-LSRTEMIETAQADTVV
Cn>KXH38191.1       L-HSHGVDAAVRSDVVDVPGGITDRN--NTGSMASD---EEKNSTH-----GKNGVVSDHEAS-LSRTEMIETAQADTVV
Ch>XP_018163968.1   L-QSHGV-----NAVVDVPGGITDRT---AGSMASD---EEKSSTH-----GKNGVIAGNEAP-LSRTEMVETAQGDTVV
Cgr>XP_008090406.1  L-RSHGV-----NAVIDVPGGIADRT---IGSMASD---EEKSSTH-----GKNGVIAKNDAP-LSGAEMIETAQGDTVV
Cgl>EQB59180.1      L-QSHGVVDAS-PDVVDVPGGIADRKETAAGSMVSD---EEKRSNHSSNKNGQHGVLGDHEVR-LSRDEMVETARGDTVV
Vd>XP_009649047.1   L-QGHGIQENVLADVVDVPGGIADRHS-TPSPTAFD---SEKNSTN----DNKNTSKFENEAT-ISRDEMLVTAQGETIL
Mo>XP_003710887.1   VGQASTTDATNQDKIVDVPGSITDKGPNASNSSEGNSFVEEKEKTRK-EKDEQVGELLDAEAGRVIKSDDAAVQNTDTIA
Consensus           : :.            ::****.*:*:      ..   :    .**  ...       :         :.  :   :    . .  :*:

                           330       340       350       360       370       380       390       400
                    ....|....|....|....|....|....|....|....|....|....|....|....|....|....|....|....|
Ca>CaNRT2.1         NPSFKEAMQVVFSPQTIFHVATYGCSFGGELAINAILSSYYLKNFPSLGQTGASNWAAMFGFLNVITRPAGGVVSDLLYR
Cn>KXH38191.1       NPSFKEAMQVVFSPQTIFHVATYGCSFGGELAINAILSSYYLKNFPSLGQTGASNWAAMFGFLNVVTRPAGGVVSDLLYR
Ch>XP_018163968.1   NPTFKDAMKVVFSPQTVFHVATYGCSFGGELAINSILSSYYLKNFPSLGQTGASNWAAMFGFLNIITRPAGGVVSDLLYR
Cgr>XP_008090406.1  NPTFKDAMKVVLSPQTLFHVATYGCSFGGELAINSILSSYYLKNFPSLGQTGASNWAAMFGFLNIITRPAGGVVSDLLYR
Cgl>EQB59180.1      NPTFKEAMSVVLSPQTIFHIATYGCSFGGELAVNAVLSSYYLKNFPALGQTGASNWAAMFGFLNVITRPAGGVVSDLLYR
Vd>XP_009649047.1   KPTFKDGLKVAISPQTIFHMATYACSFGGELAINSILSSYYLKNFPSLGQTGASNWAAMFGFLNVVTRPLGGIVGDVLYH
Mo>XP_003710887.1   KPTFGESLRVMASPQTLVHVLTYFCSFGGELAINAILSSYYLKNFPELGQTGASNYAAIFGFLNFITRPLGGVVSDLLYN
Consensus           :*:*  :.: *   ****:.*: ** ********:*::*********** ********:**:*****.:***  **:*.*:**.

                           410       420       430       440       450       460       470       480
                    ....|....|....|....|....|....|....|....|....|....|....|....|....|....|....|....|
Ca>CaNRT2.1         FGG---QNLWLKKGWITTCGVLTGALLILIGQLNPHNESTMFGLVFLMAVFHEAGNGANFGLIPHVHSYANGIVSGVTGA
Cn>KXH38191.1       YGG---QNLWLKKGWITTCGVLTGALLILIGQLNPHNESTMFGLVFLMAVFHEAGNGANFGLIPHVHSYANGIVSGVTGA
Ch>XP_018163968.1   YGG---HNLWLKKGWITVCGLLTGALLILIGQINPHKEATMFGLVFLMAVFHEAGNGANFGLIPHVHGHANGIVSGVTGA
Cgr>XP_008090406.1  FGG---HSLWLKKGWITVCGILTGALLILIGQLNPHNEATMFGLVFLMAIFLEAGNGANFGLIPHVHSHANGIVSGLTGA
Cgl>EQB59180.1      YGG---QNLWLKKAWITVCGVLTGALLLLIGLLNPHDEATMFGLIFLMAVFHEAGNGANFGLIPHVHGYANGIVSGVTGA
Vd>XP_009649047.1   FGK---QNLWLKKGWVSFCGIMTGLLLILIGRLNPHDEATMFGLIFLMAVFHEAGNGANFALVPHVHPQANGIVSGLTGA
Mo>XP_003710887.1   AAGSGPRGLWLKKGWIHVCGIATGALLILIGQLNPHHQPTMLGLVIFMAFFHEAGNGANFALIPHVHPHANGLVSGITGA
Consensus            .    :.*****.*:  **: ** **:*** :***.:.**:**::**.* ********.*:****   ***:***:***

                           490       500       510       520       530
                    ....|....|....|....|....|....|....|....|....|....|....   ID     COV
Ca>CaNRT2.1         GGNFGGVIFAIIFRFMDQGTNYAKGFWVIGCIHIAVNLLVSWIHPLPKAQRH--   100%   100%
Cn>KXH38191.1       GGNFGGVIFAIIFRFMDQGTNYAKGFWVIGCIHIAVNLLVSWIHPLPKAQRH--   99%    100%
Ch>XP_018163968.1   GGNFGGVIFAIIFRFMDNGTNYAKGFWVIGCIHIAVNLGVSWIHPLPKNQRH--   91%    100%
Cgr>XP_008090406.1  GGNFGGVVFAIIFRFMDHGANYAKGLWVIGCIHIAVYIGVSWIHPLPKNQRH--   87%    100%
Cgl>EQB59180.1      GGNLGGVIFAIIFRFMDNGTNYAKGFWVIGCIHIGVNLLVSWIHPLPKSQRH--   89%    100%
Vd>XP_009649047.1   GGNLGGIIFAIIFRFMDNGTNYAKGFWVIGCMHIGLNVLVSWIPPLPKGQIGGR   75%    99%
Mo>XP_003710887.1   GGNLGGVVFAVVFRFVGGGTGYATGFWIIGIVHIAINIAMAWIKPLPKGQIGGY   65%    99%
Consensus           ***:**::**::****:***:.  *:.**.*:*:** :**.:  :  ::** ****  *
```

Figure S5. Amino acid sequence alignment of CaNRT2.1 homologs. Cn, *Colletotrichum nymphaeae* SA-01 (KXH38191.1); Ch, *Colletotrichum higginsianum* IMI 349063 (XP_018163968.1); Cgr, *Colletotrichum graminicola* M1.001 (XP_008090406.1); Cgl, *Colletotrichum gloeosporioides* Cg-14 (EQB59180.1); Vd, *Verticillium dahliae* VdLs.17 (XP_009649047.1); Mo, *Magnaporthe oryzae* 70-15 (XP_003710887.1). ID, identity; COV, coverage. ID and COV are displayed in the blast results obtained using BlastP against the NCBI protein database.

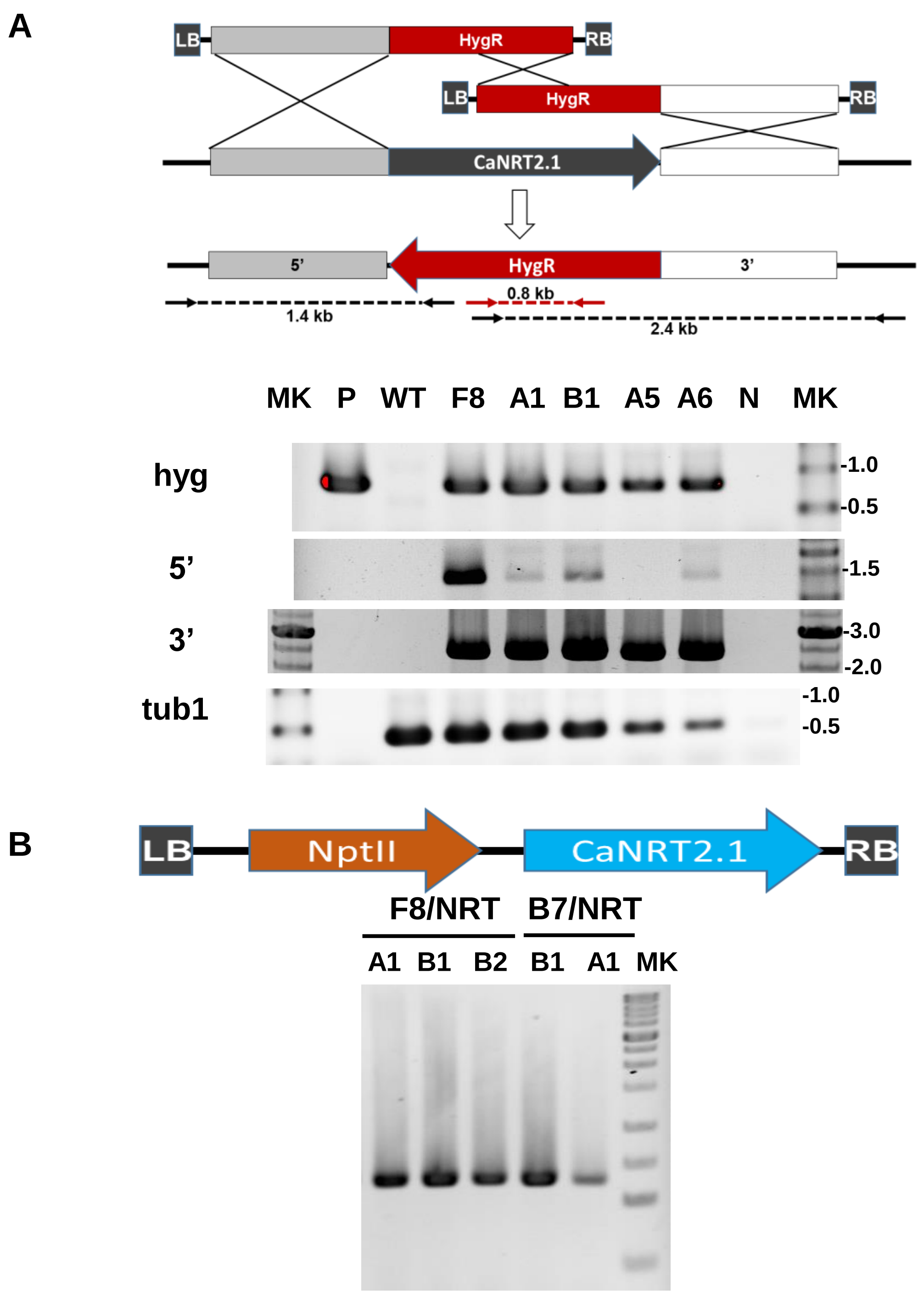

Figure S6. PCR screening for CaNRT2.1 gene replacement mutants (A), CaNRT2.1 complementation transformants of the B7 strain and ΔCaNRT2.1 strain F8 (B). A, Schematic representation of gene replacement with three homologous recombination events using the split marker strategy (upper panel) and PCR assays to confirm that three crossover events occurred in potential CaNRT2.1 gene replaced mutants (F8, A1, B1, A5 and A6). MK, DNA molecular marker; P, plasmid carrying the *hptII* cassette as a positive control of hptII crossover and a negative control of 5' and 3' crossover; WT, wild-type strain; N, negative control; tub1 used as the PCR control. Dashed lines indicate the amplified regions of the three crossover fragments. B, PCR amplification of the nptII gene for CaNRT2.1 complementation transformants in B7 and ΔCaNRT2.1 strain F8.

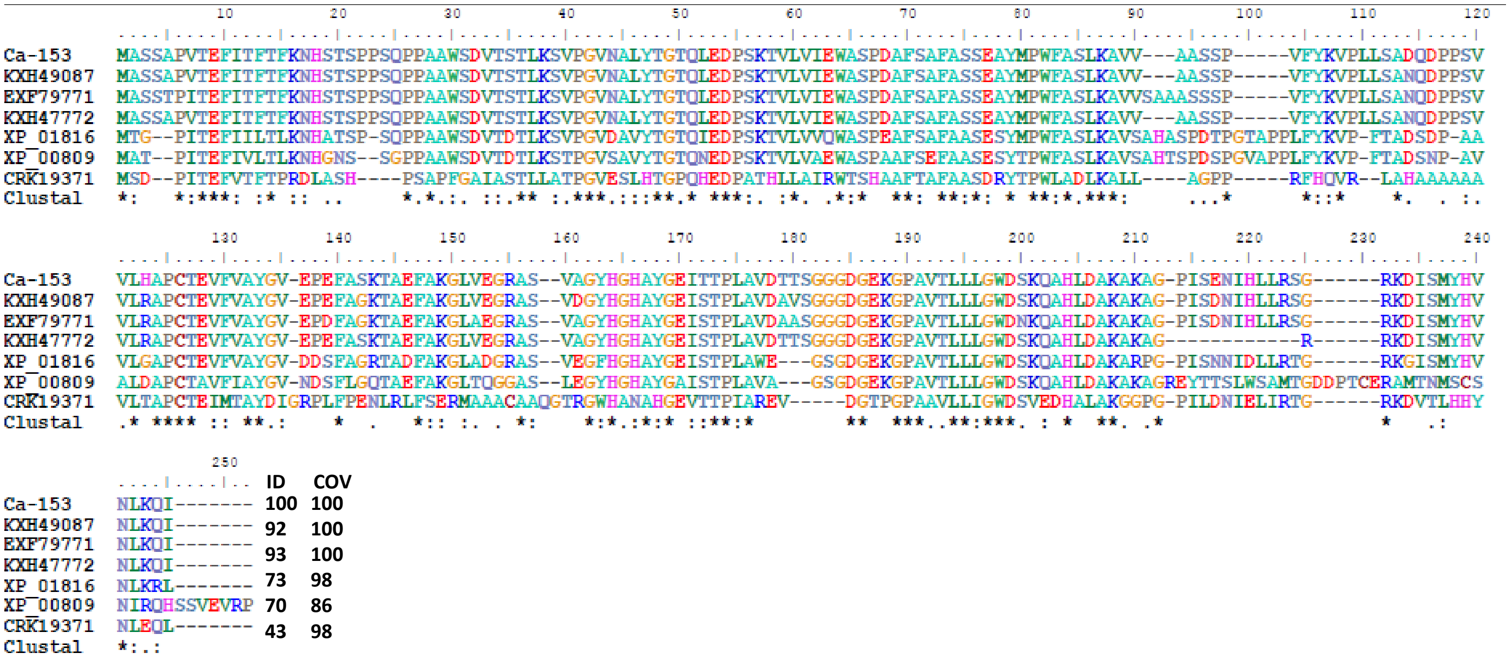

Figure S7. Amino acid sequence alignment of CaHP1 homologs. Cs, *Colletotrichum simmondsii* (KXH49087.1); Cf, *Colletotrichum fioriniae* PJ7 (EXF79771.1); Cn, *Colletotrichum nymphaeae* SA-01 (KXH47772.1); Ch, *Colletotrichum higginsianum* IMI 349063 (XP_018163966.1); Cgr, *Colletotrichum graminicola* M1.001 (XP_008090403.1); Vl, *Verticillium longisporum* (CRK19371.1). ID, identity; COV, coverage. ID and COV are displayed in the blast results obtained using BlastP against the NCBI protein database.

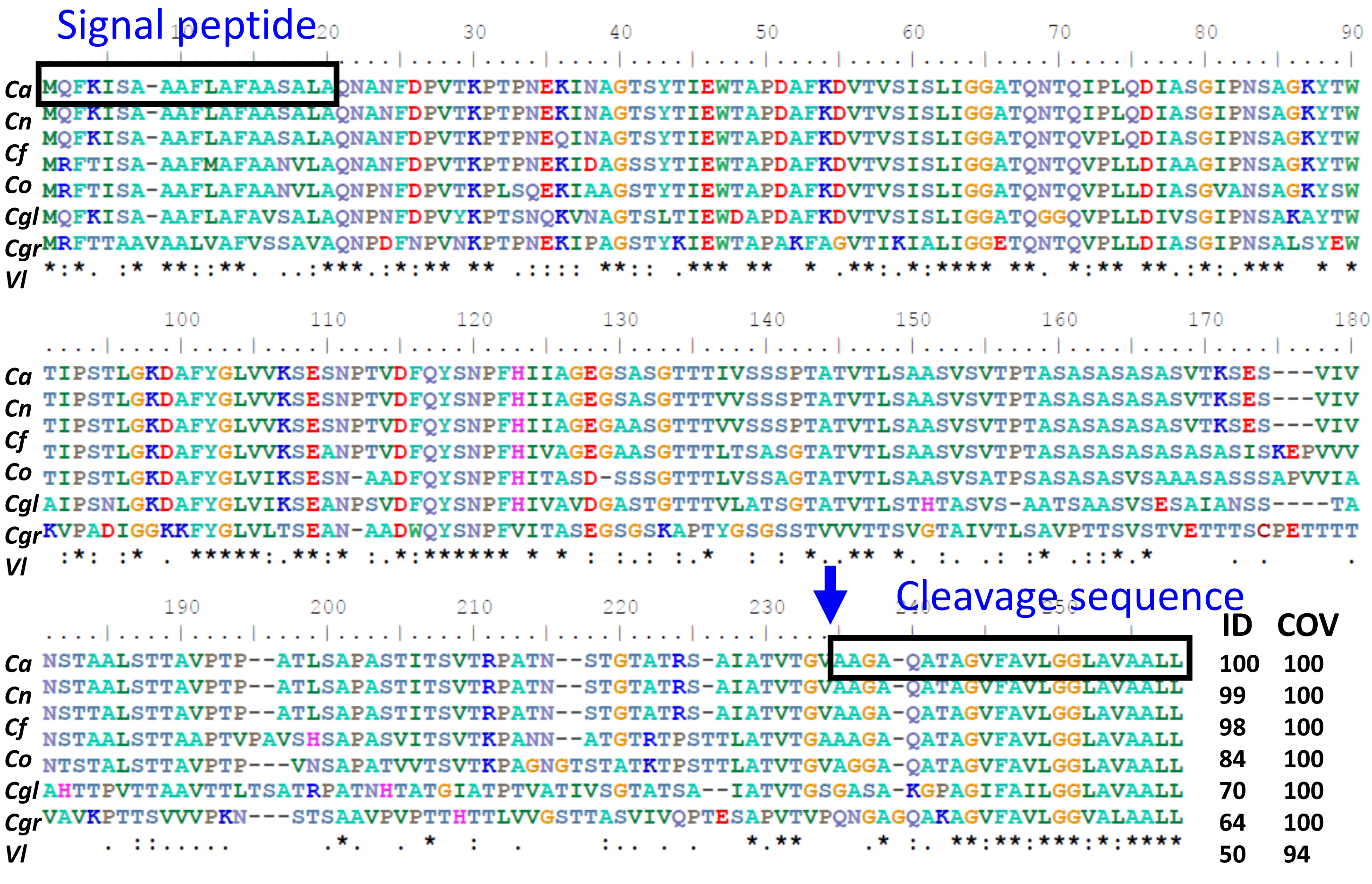

Figure S8. Amino acid sequence alignment of CaGpiP1 homologs. Signal peptides and cleavage sequences are indicated with boxes, while the ω site is indicated with an arrow. Ca, CaGpiP1; Cn, *Colletotrichum nymphaeae* SA-01 (KXH47773.1); Cf, *Colletotrichum fioriniae* PJ7 (EXF79770.1); Co, *Colletotrichum orbiculare* MAFF 240422 (TDZ25850.1); Cgl, *Colletotrichum gloeosporioides* Cg-14 (EQB59181.1); Cgr, *Colletotrichum graminicola* M1.001 (XP_008090404.1); Vl, *Verticillium longisporum* (CRK19369.1). ID, identity; COV, coverage. ID and COV are displayed in the blast results obtained using BlastP against the NCBI protein database.

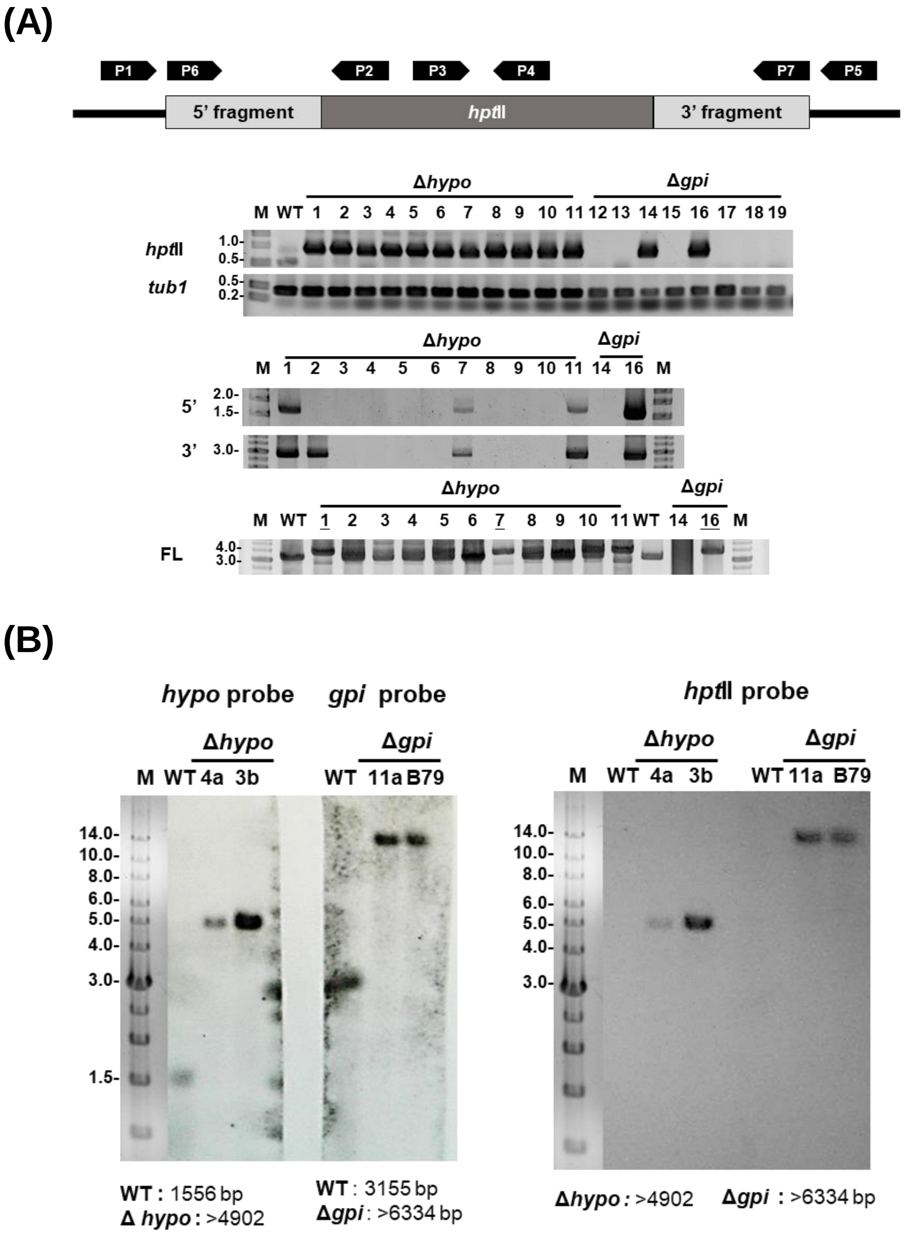

Figure S9. Screening of CaHP1 and CaGpiP1 gene knockout mutants by PCR (A) and Southern blot (B) analysis. (A) Selection marker gene (*hpt*II), 5’ and 3’ recombination fragments were amplified with primer set P3 and P4, P1 and P2 primers, and P3 and P5, respectively. The full length (FL) of the gene replaced fragment was amplified by primer set P1 and P5. Tubulin1 gene (*tub1*) was used as the positive control in PCR. (B) Southern blot analysis was performed using *CaHP1*, *CaGpiP1* or *hpt*II as the probe. The expected DNA sizes in the Southern bolting assays for the wild type and mutants hybridized with the three probes are indicated below the blotting.

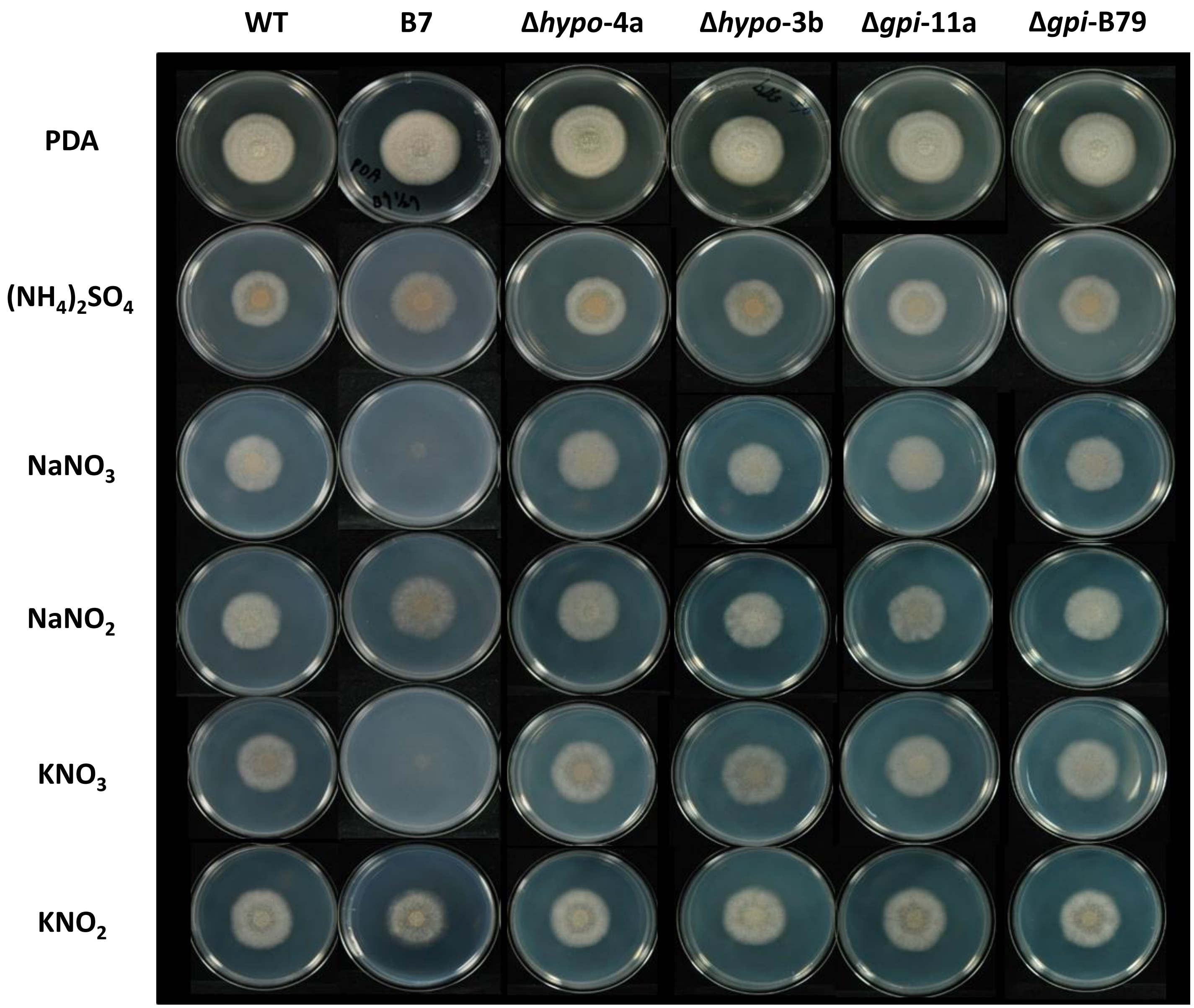

Figure S10. Colony morphology of the *Colletotrichum acutatum* wild-type (WT) strain, CaHP1 mutants (Δhypo-4a, Δhypo-3b), CaGpiP1 mutants (Δgpi-11a, Δgpi-B79), and transformant B7 on PDA and Czapek's medium containing different nitrogen sources at 5 days postinoculation.

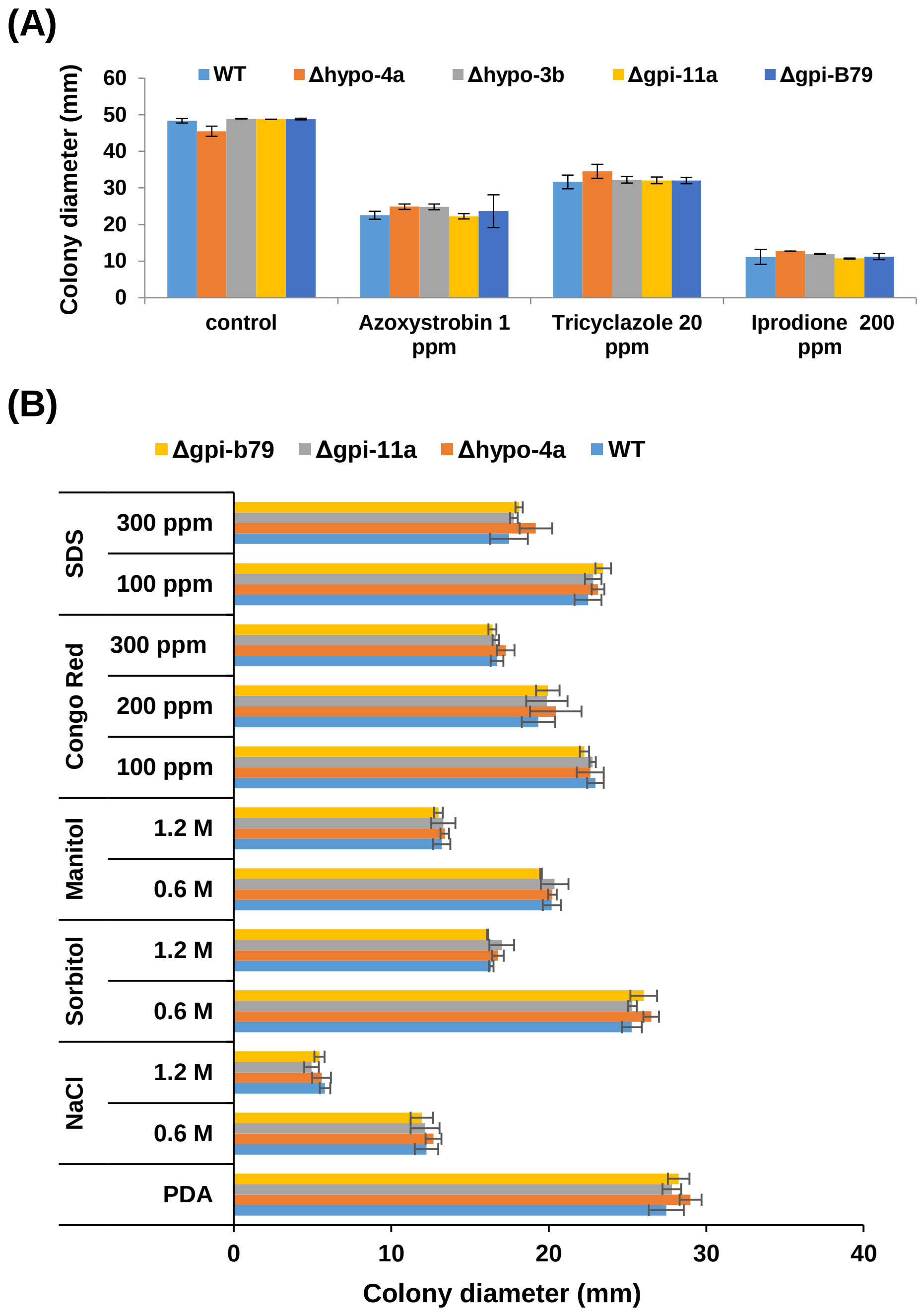

Figure S11. Growth of *Colletotrichum acutatum* Coll-153 strain (WT), CaHP1 mutants (Δ*hypo*-4a, Δ*hypo*-3b), and CaGpiP1 mutants (Δ*gpi*-11a, Δ*gpi*-B79) under different stresses. A, colony diameter of fungal strains on PDA supplemented with azoxystrobin, tricyclazole or iprodione at 7 days postinoculation.B, colony diameter of fungal strains on PDA amended with NaCl, mannitol, sorbitol, Congo red or SDS at 5 days postinoculation.
