# Supplementary material for: CaNRT2.1 Is Required for Nitrate but Not Nitrite Uptake in Chili Pepper Pathogen Colletotrichum acutatum
Source: Front Microbiol. 2021 Jan 5;11:613674. doi: 10.3389/fmicb.2020.613674 (PMC7813687; doi:10.3389/fmicb.2020.613674)
Supplement: Supplementary Table 1 — Primers used in this study. [file Data_Sheet_2.pdf]

Supplementary Tables

Table S1. Primers used in this study.

Primer name	Sequence (5' → 3')	Purpose
HygR2/lac-pro	GTCTGGACCGATGGCTGTG<br>ACTTTATGCTTCCGGCTCGTAT	Inverse PCR for T-DNA insertion site identification.
LB2/M3-reverse	CATGTGTTGAGCATATAAGAAACCCT<br>CAGGAAACAGCTATGAC	Nested PCR for T-DNA insertion site identification.
LB3/T3	GAATTAATTCGGCGTTAATTCAGT<br>AATTAACCCTCACTAAAGGG	Nested PCR for T-DNA insertion site identification.
Primer set 1 (B7-gpi-RNA-F/R)	TCCTACACCATCGAGTGGACTG<br>ACAGCGAAGACACCAGCAGTAG	Deletion fragment amplification; Semi-q-rt-PCR of CaGpiP1.
Primer set 2 (B7-hypo-RNA-F/R)	CGTAACATCGACCCTCAAATCC<br>GTCGAGGTGAGCCTGCTTAC	Deletion fragment amplification; Semi-q-rt-PCR of CaHP1.

	TA	
Primer set 3<br>(B7-gpi-3'F/R)	ggtGAGCTCGCAACGTCCGTT<br>GCAGCATTTACT<br>ggtAAGCTTAGACAGCAGAGA<br>TGTTGTCGTAGC	Deletion fragment amplification.
Primer set 4<br>(B7-gpi-5'F/R)	ggtGAGCTCGCTTCAATTCAC<br>GCTGCAGTCAAG<br>gtggtGAATTCTGTGATGGATT<br>GAGTCGACGAGGT	Deletion fragment amplification
Primer set 5<br>(B7-tDNA-<br>F1/R1)	ATGCCCGAAACTTGGCTCTT<br>GTTG<br>GCATTACACTATTGGCGTCTC<br>CTG	Deletion fragment amplification.
Primer set 6<br>(B7-hypo-5'-R/F)	gtggtGAATTCTGTTTGTGACC<br>CGGTTGACTTTGG<br>gtggtGAATTCTGTTTGTGACC<br>CGGTTGACTTTGG	Deletion fragment amplification.
Primer set 7<br>(Ca-NTP-sq-F/R)	AACAAGCCTCGAGCTTCGTA<br>ATCC<br>GTCCGTTCCATACTCCAGAC	Deletion fragment amplification.

	AGAC	
Primer set 8 (Ca-NTP-RNA-F/R)	AGGTGAACCCTATCAACCGCAAAG<br>GAAGCAGCGAGCCAAAGACGATAC	Deletion fragment amplification.
Primer set 9 Ca-NPT-RNA-F/Ca-NPT-sq-R2)	AGGTGAACCCTATCAACCGCAAAG<br>GTTGAGGAAGCCAAACATGGCAGC	Deletion fragment amplification.
Primer set 10 (Ca-NTP-3'-F-XbaI/Ca-NTP-3'-ck-R)	GTtctagaCGCAGGGACGTTCACGAACATTAA<br>ATTCGTCGTTGGTGTTTACACCCG	Deletion fragment amplification.
Primer set 11 (RecQ-F/R)	CACCTCTTCACAGACGCAAAAGTC<br>GGCCTCTTATTGTTTCGGCAGATG	Deletion fragment amplification.
Primer set 12 (RecQ-3'-F/R)	CCTACCATTCCCGTGAGATATCAG<br>AGCTTCACTCATTTGCCGAC	Deletion fragment amplification.

	AGAG	
M13-r/RecQ-3’-R	AGCTTCACTCATTTGCCGAC AGAG	Deletion fragment amplification.
M13-r/ NTP-3’ck-R	ATTCGTCGTTGGTGTTTACAC CCG	Deletion fragment amplification.
Tub1-F/R	ACCATTCACCCTGAGCATGG<br>TGACCCTTTGCCCAGTTGTT	PCR control of DNA deletion fragment amplification.
pTrpC – EcoRI/XhoI-5'-Hyg	GGTGGGAATTCTGATATTGA AGGAGCATTTTTTGGGC<br>TGGTCTCGAGGGGGCAGTCC TCGGCCC	5'-hyg cassette amplification for p1300-Hyg5 construction.
3'-Hyg-XhoI/ EcoRI-3'-Hyg	TGGTCTCGAGGTCTGTCGAG AAGTTTCTGATC<br>GGTGGGAATTCCTATTCCTTT GCCCTCGGAC	3'-hyg cassette for p1300-Hyg5 construction.
HygR3<br>HygR5	GGATGCCTCCGCTCGAAGTA<br>CTTAAGTTCGCCCTTCCTCC	Transformant screening for hptII recombination; Probe preparation.
OligodTV	TTTTTTTTTTTTTTTTTV	cDNA synthesis.

nptII-1<br>nptII-2	AATATCACGGGTAGCCAACG<br>AGACAATCGGCTGCTCTGAT	Gene complementation vector construction.
tub1_F2<br>tub1_R2	ACCATTCACCCTGAGCATGG CTTG<br>AGTGGCCCTTTGCCCAGTTG TTAC	Semi-RT-PCR for tubulin gene.
Ca_NTP_5'_ck_F<br>Ca_NTP_3'_ck_R	TACACCACCCCGTTTATTTCT GGC<br>ATTCGTCGTTGGTGTTTACAC CCG	CaNRT2.1 gene knockout confirmation.
B7_hypo RNA _F<br>B7_hypo RNA _R	CGTAACATCGACCCTCAAAT CC<br>GTCGAGGTGAGCCTGCTTAC TA	Semi qRT-PCR for CaHP1.
B7_gpi RNA _F<br>B7_gpi RNA _R	TCCTACACCATCGAGTGGAC TG<br>ACAGCGAAGACACCAGCAG TAG	Semi qRT-PCR for CaGpiP1.
B7_hypo_5'F (SacI)	GGTGAGCTCATCTGGTGTCA CAAGACCGCCTTT	Amplification of CaHP1 5’ flanking sequence for gene

B7_ hypo_5'R (EcoRI)	GTGGTGAATTCTGTTTGTGA CCCGGTTGACTTTGG	disruption; Probe preparation for Southern blotting.
B7_ hypo _3'F (EcoRI)<br>B7_ hypo_3'R (SacI)	GTGGTGAATTCGACTGACGA TGGACAGTATCACGT<br>GGTGAGCTCAAGATCAACGA GCCGCAAGACAAC	Amplification of CaHP1 3' flanking sequence for gene disruption.
B7_gpi_5'F (SacI)<br>B7_gpi_5'R (EcoRI)	GGTGAGCTCGCTTCAATTCA CGCTGCAGTCAAG<br>GTGGTGAATTCTGTGATGGA TTGAGTCGACGAGGT	Amplification of CaGpiP1 5' flanking sequence for gene disruption; Probe preparation for Southern blotting.
B7_gpi_3'F (SacI)<br>B7_gpi_3'R (HindIII)	GGTGAGCTCGCAACGTCCGT TGCAGCATTTACT<br>GGTAAGCTTAGACAGCAGAG ATGTTGTCGTAGC	Amplification of CaGpiP1 3' flanking sequence for gene disruption.
Ca_NTP_5'_F_Hi ndIII<br>Ca_NTP_5'_R_X baI	GGTAAGCTTCGCACATGCCA TCTATGGTCGAAT<br>GTTCTAGAGCGTTGTTTCCTC GTAGTTTTCGG	Amplification of CaNRT2.1 5' flanking sequence for gene disruption
Ca_NTP_3'_F_X baI	GTTCTAGACGCAGGGACGTT CACGAACATTAA	Amplification of CaNRT2.1 3' flanking sequence for gene

Ca_NTP_3'_R_HindIII	GGTAAGCTTGGCCTACTTGACGACGACATTTCT	disruption
Hypo5’F-ck<br><br>Hyg1	CACATCGCTACGTACTACGTCG<br><br>CACAAATCGCCCGCAG AA	Amplification of CaHP1 gene 5’ flanking cross-over
Hypo3’R-ck<br><br>HyR3	GACCTGCCACTACATTCAAGCG<br><br>GGATGCCTCCGCTCGAAGT	Amplification of CaHP1 gene 3’ flanking cross-over.
gpi5’F-ck<br><br>Hyg1	TGCCTCGAAGGTGGTACTGTTG<br><br>CACAAATCGCCCGCAG AA	Amplification of CaGpiP1 gene 5’ flanking cross-over.
gpi3’R-ck<br><br>HyR3	GCATACTCCCACAACGTTCCTC<br><br>GGATGCCTCCGCTCGAAGT	Amplification of CaGpiP1 gene 3’ flanking cross-over.
GFP_XmaI_ApaI_XhoI_ F2<br><br>GFP_nosT_HindIII_R2	TCCCGGGGGCCCCTCGAGATGGTGAGCAAGGGCGAGGA<br><br>GGTAAGCTTCGGATCTAGTAACATAGATGACACCGCGC	pPgpd-GFP(I) construction

GFP_BamHI_XhoI_ F<br>GFP del-taa_XmaI_ApaI_R	GGTGGATCCCTCGAGATGGTGAGCAAGGGCGAGGA<br>GCCCGGGGGGCCCCTTGTACAGCTCGTCCATGC	pPgpd-GFP(II) construction
gpi-F-SpeI<br>gpi-del TAA-R-XmaI	GGTACTAGTATGCAGTTCAAGATCTCCGCCG<br>TCCCGGGGAGAAGGGCAGCAACGGCGA	GPI-GFP-I construction
mgpi-F-XmaI<br>Tgpi-R-HindIII	TCCCGGGCAGAATGCCAACTTCGACCCCGT<br>GGTAAGCTTGAGGCAGTAGTGAGACGGAATATC	partial CaGpiP1 ORF amplification for GPI-GFP-III construction
gpi-F-SpeI<br>gpi-delCS- R-XhoI	GGTACTAGTATGCAGTTCAAGATCTCCGCCG<br>GGTGCTCGAGAACACCAGTGACGGTGGCGATAGC	partial CaGpiP1 amplification for GPI-GFP-II construction
gpi-CS-F-XmaI<br>Tgpi-R-HindIII	TCCCGGGGCTGCTGGTGCCCAGGCTACTGCT<br>GGTAAGCTTGAGGCAGTAGT	cs fragment amplification for GPI-GFP-II construction

	GAGACGGAATATC	
B7_hypo2_F_XmaI<br>B7_hypo2_R_XbaI	TCCCGGGTTGACCTGCCACTACATTCAAGCG<br>GGTGGTTCTAGACCATCCTCGTAGCTTTCTTCTTCC	CaHP1 complementation
B7_GPi_F2_XmaI<br>B7_Gpi_R2_XbaI	TCCCGGGAGAGCTTTGGTTGCCTCGAAGGTG<br>GGTGGTTCTAGAGAATCCGAGTAAATGCTGCAACGG	CaGpiP1 complementation
Ca-NTP-5’-XmaI-F<br>Ca-NTP-3’-XbaI-R	TCCCGGGCGCACATGCCATCTATGGTCGAAT<br>GTCTAGAGTCCGTTCCATACTCCAGACAGAC	CaNRT2.1 complementation

Table S2. Plasmids used in this study.

**Plasmid ID**	**Description**	**Backbone vector**
p1300-Hyg5'	T-DNA carrying 5’ fragment of hptII cassette	pCAMBIA1300
p1300-Hyg3'	T-DNA carrying 3’ fragment of hptII cassette	pCAMBIA1300
p1300-Hyg3’- hypo5’	3’ hptII cassette ligated to 5’ flanking of CaHP1 gene	p1300-Hyg3'
p1300-Hyg5’- hypo3’	5’ hptII cassette ligated to 3’ flanking of CaHP1 gene	p1300-Hyg5'
p1300-Hyg3’- GPI5’	3’ hptII cassette ligated to 5’ flanking of CaGpiP1 gene	p1300-Hyg3'
p1300-Hyg5’- GPI3’	5’ hptII cassette ligated to 3’ flanking of CaGpiP1 gene	p1300-Hyg5'
p1300-Hyg3' - NTP5'	3’ hptII cassette ligated to 5’ flanking of CaNRT2.1	p1300-Hyg3'
p1300-Hyg5' - NTP3'	5’ hptII cassette ligated to 3’ flanking of CaNRT2.1	p1300-Hyg5'
pN1300	Complementation vector	PCAMBIA1300
pN1300-NTP	Complementation of CaNRT2.1	pN1300

pPgpdG	GFP driven by Pgpd constitutive promoter	pBHt2-Pgpd
pPgpdmidG	GFP without stop-codon (taa) driven by Pgpd constitutive promoter	pBHt2-Pgpd
pGPI-GFP-I	Pgpd::GPI::eGFP	pPgpdG
pGPI-GFP-II	Pgpd::mGPI::eGFP del taa::CS	pPgpdmidG
pGPI-GFP-III	Pgpd::sp::eGFP del taa::mGPI	pPgpdmidG

Table S3. The *p* values of the statistical analysis of the lesion size caused by gene knockout strains of CaHP1 (Δ*hypo*-4a and Δ*hypo*-3b) and CaGpiP1 (Δ*gpi*-11a and Δ*gpi*-B79) or transformant B7 on the fruits of chili pepper, tomato and bell pepper compared with the wild-type strain.

strain	*p* value of paired *t*-test			
	Chili pepper Exp.1	Chili pepper Exp. 2	Tomato	Bell pepper
Δ*hypo*-4a	0.62	0.42	0.32	0.50
Δ*hypo*-3b	0.51	0.64	0.98	UD
Δ*gpi*-11a	0.07	0.86	0.68	UD
Δ*gpi*-B79	0.52	0.33	0.35	0.25
B7	0.12	UD	0.32	UD

UD, Undetected.
